# Clinically Translatable Solid‐State Dye for NIR‐II Imaging of Medical Devices

**DOI:** 10.1002/advs.202303491

**Published:** 2023-11-09

**Authors:** Deling Li, Hui Shi, Qingrong Qi, Baisong Chang, Yuanwen Jiang, Kun Qian, Xiudong Guan, Peng Kang, Ning Ma, Yuan Zhang, Zeyu Zhang, Xiaojing Shi, Chunrong Qu, Yilei Wu, Weiyu Chen, Hao Chen, Baowang Li, Liangpeng Chen, Ziyang Li, Shunchang Ma, Lingyun Xu, Yanrong Zhang, Jie Tian, Zhenhua Hu, Wang Jia, Zhen Cheng

**Affiliations:** ^1^ Department of Neurosurgery Beijing Tiantan Hospital China National Clinical Research Center for Neurological Diseases Beijing Neurosurgical Institute Capital Medical University Beijing 100070 China; ^2^ Molecular Imaging Program at Stanford (MIPS) Bio‐X Program, and Department of Radiology Stanford University Stanford CA 94305 USA; ^3^ State Key Laboratory of Drug Research Molecular Imaging Center Shanghai Institute of Materia Medica Chinese Academy of Sciences Shanghai 201203 China; ^4^ Shandong Laboratory of Yantai Drug Discovery Bohai Rim Advanced Research Institute for Drug Discovery Yantai Shandong 264117 China; ^5^ Institute of Molecular Medicine College of Life and Health Sciences Northeastern University Shenyang 110000 China; ^6^ West China School of Pharmacy Sichuan University Chengdu 610041 China; ^7^ State Key Laboratory of Advanced Technology for Materials Synthesis and Processing Wuhan University of Technology Wuhan 430070 China; ^8^ Department of Chemical Engineering Stanford University Stanford CA 94305 USA; ^9^ Interventional Neuroradiology Center Beijing Tiantan Hospital Capital Medical University Beijing 100070 China; ^10^ CAS Key Laboratory of Molecular Imaging Beijing Key Laboratory of Molecular Imaging The State Key Laboratory of Management and Control for Complex Systems Institute of Automation Chinese Academy of Sciences Beijing 100190 China; ^11^ School of Artificial Intelligence University of Chinese Academy of Sciences Beijing 100049 China; ^12^ Beijing Advanced Innovation Center for Big Data‐Based Precision Medicine School of Medicine Beihang University Beijing 100191 China

**Keywords:** aggregates, in vivo imaging, medical devices, NIR‐II fluorescence

## Abstract

Medical devices are commonly implanted underneath the skin, but how to real‐time noninvasively monitor their migration, integrity, and biodegradation in human body is still a formidable challenge. Here, the study demonstrates that benzyl violet 4B (BV‐4B), a main component in the FDA‐approved surgical suture, is found to produce fluorescence signal in the first near‐infrared window (NIR‐I, 700–900 nm) in polar solutions, whereas BV‐4B self‐assembles into highly crystalline aggregates upon a formation of ultrasmall nanodots and can emit strong fluorescence in the second near‐infrared window (NIR‐II, 1000–1700 nm) with a dramatic bathochromic shift in the absorption spectrum of ≈200 nm. Intriguingly, BV‐4B‐involved suture knots underneath the skin can be facilely monitored during the whole degradation process in vivo, and the rupture of the customized BV‐4B‐coated silicone catheter is noninvasively diagnosed by NIR‐II imaging. Furthermore, BV‐4B suspended in embolization glue achieves hybrid fluorescence‐guided surgery (hybrid FGS) for arteriovenous malformation. As a proof‐of‐concept study, the solid‐state BV‐4B is successfully used for NIR‐II imaging of surgical sutures in operations of patients. Overall, as a clinically translatable solid‐state dye, BV‐4B can be applied for in vivo monitoring the fate of medical devices by NIR‐II imaging.

## Graphical abstract

Benzyl Violet 4B (BV‐4B), serving as a component in the FDA‐approved surgical sutures, is first discovered as a novel solid‐state dye in the NIR‐II window (1000–1700 nm). It can be used to non‐invasively monitor the degradation process of surgical sutures, to diagnose the rupture of BV‐4B‐coated silicone catheter and to guide the hybrid fluorescence‐guided surgery (hybrid FGS) of arteriovenous malformation (AVM) with embolization glue.

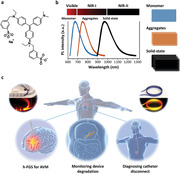

## 1 Introduction

Medical devices are commonly implanted in loose connective tissues underneath the skin, where different categories of implantable biomaterials in aggregated states have been widely used.^[^ ^1^ ^]^ Implantable medical devices, e.g., surgical sutures and catheters in ventriculoperitoneal shunt for hydrocephalus treatment, are generally composed of biodegradable and nondegradable materials.^[^ ^2^ ^]^ However, the biodegradation kinetics exists from person to person, from tissue to tissue, and varies over time.^[^ ^2b^ ^]^ Moreover, the migration, integrity damage and wear of the devices usually happen in the body of patients.^[^ ^3^ ^]^ How to continuously and noninvasively monitor the medical devices in human body is still challenging. There have been no reliable approaches, so far, to in vivo visualize and monitor the position, integrity and biodegradation processes of medical devices without radiation.^[^ ^4^ ^]^

Fluorescent luminogens with desired properties have enabled the rapid development of many research fields. Especially, the aggregated fluorophores, which display unique photophysical properties from those of monomers including red‐shifted absorption and fluorescence spectra and enhanced quantum yields, open new vital research directions.^[^ ^5^ ^]^ Most aggregated probes have their absorbance and emission in the traditional visible (400–700 nm) and the first near‐infrared window (NIR‐I, 700–900 nm). The probes in the second near‐infrared window (NIR‐II, 1000–1700 nm) are particularly appealing because of recently discovered benefits including low autofluorescence interference, deep penetration depth, and high resolution compared with short‐wavelength fluorescence probes.^[^ ^6^ ^]^ Several NIR‐II aggregated dyes based on complicated synthesis chemistries, e.g., molecular engineering method, have been successfully designed and attracted lots of research attentions.^[^ ^7^ ^]^ However, all the reported NIR‐II aggregated dyes are inherently hydrophobic and are usually co‐precipitated to form nanodots or nanoparticles to ensure water‐dispersibility,^[^ ^5^ ^]^ hindering their biological applications especially translating into clinic. Besides, until now there have been no FDA‐approved NIR‐II dyes reported in clinical use.

Organic probes have broad potential applications to solve several bottlenecks in clinical applications such as fluorescence‐guided therapy under aggregation conditions.^[^ ^8^ ^]^ Recently, polymeric coating containing NIR‐I fluorescent dyes have been deposited on catheters or other medical devices to render them visualizable.^[^ ^9^ ^]^ However, the relatively complicated luminogen production inhibits the translational application and the imaging depth limitation of NIR‐I probes further makes it feasible only in specific situations. It is conceived that, if NIR‐II organic probes with stable chemical structures can be used to fluorescently image the medical devices in depth and to monitor their status in vivo, it will largely facilitate the development of medical devices. Therefore, clinically translatable dyes that emit strong NIR‐II fluorescence in the solid state and can be easily fabricated onto medical devices are crucial, but their development remains a challenge.

**Figure 1:**
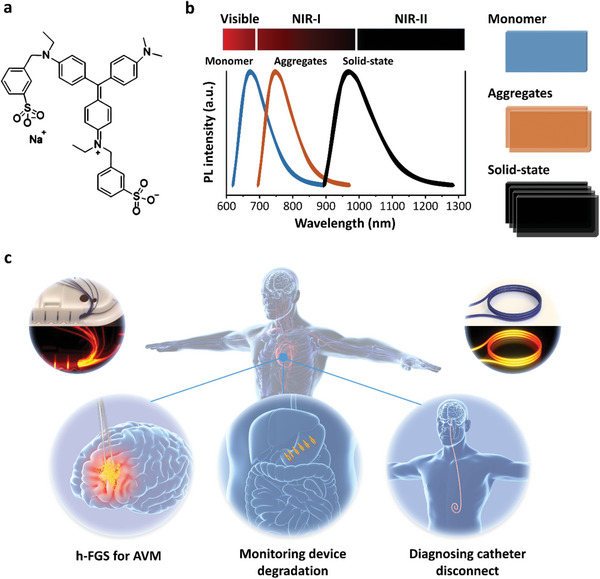
Schematic illustration of clinically translatable solid‐state dye for NIR‐II imaging of medical devices. a) Chemical structure of BV‐4B. b) Fluorescence spectra of BV‐4B in highly dispersed state, aggregated state (orange line), and solid powder (black line), respectively. Cartoons on the right panel showed typical molecular arrangements of BV‐4B in different states. c) Discovery of BV‐4B as a novel and clinical translatable solid‐state dye based on the finding that the surgical sutures coated with BV‐4B emitted strong NIR‐II fluorescence, and the translational potentials in three applicable situations. hybrid FGS: hybrid fluorescence‐guided surgery; AVM: arteriovenous malformation.

To decrease clinical translation barriers and facilitate clinical applications of NIR‐II imaging of medical devices, we have searched for suitable organic probes with potential aggregated property from those already safely used in medicine. Benzyl violet 4B (BV‐4B) has been widely coated on the biodegradable sutures and broadly used in clinic. In this study, we demonstrated that BV‐4B can emit NIR‐I fluorescence in polar solutions, while solid‐state BV‐4B self‐assembled into highly crystalline aggregates upon a formation of ultrasmall nanodots (**≈**3.0 nm) and produced strong NIR‐II signals with a dramatic bathochromic shift of **≈**200 nm absorption (**Figure** **1**a,b). In addition, biocompatible NIR‐II aggregates of BV‐4B allowed continuous visualization of biodegradation process of sutures, the wear of catheters via lighting‐up the subcutaneous implanted medical devices, and guidance of hybrid fluorescence‐guided surgery (hybrid FGS) of arteriovenous malformation (AVM) with embolization glue (Figure 1c). More importantly, this proof‐of‐concept research has been further successfully translated into a patient study with surgical sutures coated with BV‐4B due to the FDA‐approved sutures and dye.

## 2 Results

### 2.1 Photophysical Properties and Theoretical Calculation of BV‐4B

**Figure 2:**
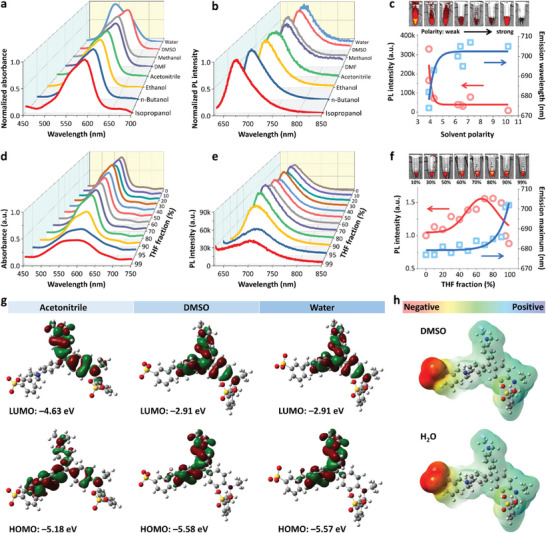
Photophysical property and theoretical calculation. a,b) Normalized a) absorption and b) emission spectra of BV‐4B in different solvents (water, DMSO, DMF, methanol, ethanol, isopropanol, and n‐butanol). Excitation wavelength: 570 nm. c) Plot of PL intensity (left) and maximum emission wavelength of BV‐4B versus a broad range of solvent polarity. Top panel: NIR‐I imaging of BV‐4B in different solutions. d,e) Normalized d) absorption and e) emission of BV‐4B in THF/methanol mixtures with different THF fractions (*f*_THF_). f) Plot of PL intensity (left) and maximum emission wavelength (right) of BV‐4B versus a broad range of *f*_THF_. Top panel: NIR‐I imaging of BV‐4B in THF/Methanol mixtures with different THF fractions (*f*_THF_). g) Calculated frontiers molecular orbital amplitude plots and electronic properties of the BV‐4B in acetonitrile (left), DMSO (middle) and water (right), respectively. h) Positive and negative charge distribution of BV‐4B in water.

The absorbance properties and photoluminescence (PL) behaviors of BV‐4B were studied. Generally, BV‐4B had good solubility in polar solvents, such as water, dimethyl sulfoxide (DMSO) and dimethylformamide (DMF) (Table S1, Supporting Information). In water solution, BV‐4B showed a strong absorption peak at ≈545 nm. The absorbance of BV‐4B dissolved in other less polar solvents exhibited a redshift of absorbance peak, e.g., 600 nm in DMSO and 595 nm in DMF, respectively (**Figure** **2**a). For methanol, ethanol, isopropanol and n‐butanol, the solubility of BV‐4B decreased and the corresponding solutions showed different colors (Figure S1, Supporting Information), while the PL intensity of BV‐4B enhanced with a decrease in maximum emission peaks (Figure 2b,c), especially in isopropanol and n‐butanol. For BV‐4B in water/DMSO solutions, it can be found a weak emission in dilute water solution but enhanced emission with increasing the DMSO volume fraction (*f*_DMSO_) from 10% to 99% (Figure S2a–c, Supporting Information). Likewise, because of the insoluble and aggregation of BV‐4B in tetrahydrofuran (THF) (Figure s3 , Supporting Information), the optical properties of BV‐4B was observed to change remarkably upon increasing the THF volume fraction(*f*_THF_) up to 99% (Figure 2d–f). For example, BV‐4B displayed a distinctly narrower, redshifted absorption and emission spectrum by varying the *f*_THF_ from 0 to 70%. The 3D spectroscopy obviously revealed BV‐4B aggregation played contribution for longer‐wavelength fluorescence emission (Figure S4, Supporting Information). After incubating at room temperature for 24 h, the PL intensity of BV‐4B enhanced dramatically (Figure S5, Supporting Information). The dynamic light scattering results indicated that BV‐4B aggregated further with incubation time (Figure S6, Supporting Information).

The intriguing optical properties encouraged us to investigate the structure characteristics of BV‐4B by theoretical calculation. First, the difference between two forms of BV‐4B in strong protic solvents and aprotic solvents was studied via density functional theory (DFT) calculation (Figure 2g,h). In acetonitrile, the electron clouds of the highest occupied molecular orbital (HOMO) were mainly located on the methylene‐cyclohexa‐2,5‐dien‐1‐imine and two phenyls units, whereas the orbitals of the lowest unoccupied molecular orbital (LUMO) were primarily localized on the dimethylaminophenyl moiety. The corresponding bandgap was calculated to be 0.55 eV, which was increased to 2.67 eV upon dissolving BV‐4B in DMSO. In water, the electron clouds of the HOMO were mainly located on the methylene‐cyclohexa‐2,5‐dien‐1‐imine and dimethylaminophenyl units, while the methylene‐cyclohexa‐2,5‐dien‐1‐imine, dimethylaminophenyl and phenyl moieties dominated the LUMO. The bandgap was estimated to be ≈2.66 eV. This finding suggested an intramolecular charge transfer characteristic of the molecules in water. Based on the bandgaps, it was speculated that the absorption spectra in acetonitrile should be longer than that in water, well consistent with optical analysis (Figure 2c).

In addition, the dihedral angles between a‐b, a‐c, and b‐c of BV‐4B (Figure S7, Supporting Information) in different solvents were calculated (Table S2, Supporting Information). The dihedral angles between a‐b, a‐c, and b‐c of BV‐4B in water, acetonitrile, methanol and THF were different, which suggested the steric configurations were different. The different steric structure resulted in totally different fluorescence mechanism.

The photophysical behavior of BV‐4B in physiological environment was examined in aqueous phosphate buffer solution (phosphate buffered saline, PBS, pH 7.4) with different volume of fetal bovine serum (FBS). BV‐4B was found to emit NIR‐I fluorescence in solution (excitation wavelength: 570 nm; emission wavelength: 700 nm). After adding FBS into the buffer solution, the color changed (Figure S8a, Supporting Information) and the emission intensity of BV‐4B enhanced dramatically (Figure S8b,c, Supporting Information). The absorbance peak of BV‐4B showed a redshift and the PL intensity enhanced with the FBS fraction increasing (Figure S9a,b, Supporting Information). BV‐4B aqueous PBS was injected subcutaneously in the footpad of the BALB/C mice. The popliteal lymph node was sequentially and clearly observed by NIR‐I imaging at 30 min after injection (Figure S9c,d, Supporting Information).

### 2.2 Characterization of BV‐4B Aggregates

**Figure 3:**
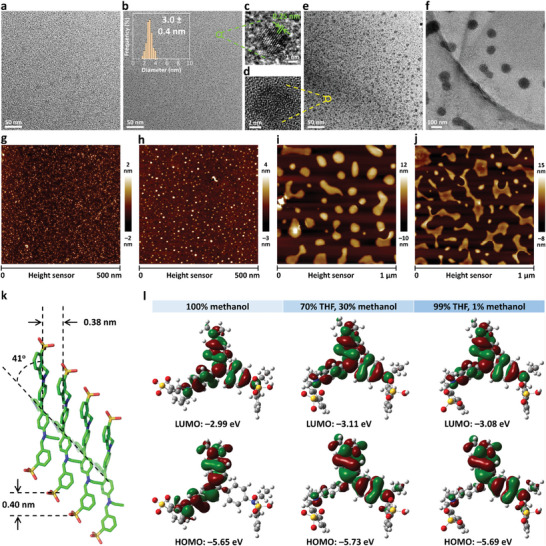
Characterizations of BV‐4B aggregates. a–f) TEM images of BV‐4B in THF/methanol mixtures with different THF fractions from a) 0%, b) 70%, e) 80% to f) 95%, respectively. Inset in (b) the corresponding size distribution histogram of BV‐4B nanodots by measuring over 100 particles. c,d) HR‐TEM images of BV‐4B in THF/methanol mixtures with THF fractions of c) 70% and d) 90%, respectively. g–j) AFM images of BV‐4B in THF/methanol mixtures with different THF fractions from g) 50%, h) 70%, i) 95% to j) 99%, respectively. k) Molecular packing diagram of BV‐4B. Solvent molecules and H atoms are omitted for clarity. l) Calculated frontiers molecular orbital amplitude plots and electronic properties of the BV‐4B in THF/methanol mixtures with different THF fractions.

The structural properties of BV‐4B aggregates were next characterized. In sharp contrast with the transmission electron microscopy (TEM) image of BV‐4B in pure methanol, the aggregates of BV‐4B in THF/methanol mixture with 70% THF fraction showed the formation of spherical‐shaped nanoparticles with a relatively narrow size distribution with a mean diameter of 3.0 nm (**Figure** 3a,b). Moreover, the size of BV‐4B aggregates remarkably increased with the change of THF fractions from 80% to 95% (Figure 3e,f). Very interestingly, a high‐resolution TEM (HR‐TEM) image of the BV‐4B nanodots at 70% THF fraction clearly revealed lattice fringes with ≈0.25 nm lattice spacing, whereas the crystalline structures will be lost when BV‐4B was dissolved in 80% THF fraction (Figure 3c,d). The aggregation process was time‐ and temperature‐dependent (Figure S10, Supporting Information). These findings were consistent with the spectroscopic properties of BV‐4B and strongly suggested the formation of ultrasmall BV‐4B nanodots, in which the BV‐4B molecules formed strongly coupled aggregates. The type of molecular packing was further investigated by single‐crystal structure analysis. The neighboring BV‐4B molecules showed clear slipping with a distance of ≈0.40 nm (Figure 3k). The intermolecular distance was ≈0.38 nm, which confirmed its π−π stacking character.^[^ ^7b^ ^]^ Meanwhile, the slip angle was estimated to be 41°. Taken together, these results indicated that aggregates with ordered molecular arrangement were produced in the ultrasmall BV‐4B nanodots.

We next carried out the theoretical calculation to understand the different absorption and emission properties of BV‐4B aggregate. Figure 3l presented the graphic demonstrations of the HOMOs and LUMOs of BV‐4B molecules in mixed solvents with different polarities. It was obvious that the HOMOs were delocalized through most of the conjugation backbones. Energy levels (*E*) of the frontier orbitals were also calculated to find the energy gaps (Δ*E*) between HOMOs and LUMOs, which were 2.66, 2.62, and 2.61 eV for BV‐4B in 100%, 30%, and 1% methanol, respectively. The value of Δ*E* decreased as the polarity of the solvent reduced, and correspondingly, the fluorescent emission wavelength would increase, which was in accordance with optical properties in Figure 2f.

### 2.3 Strong NIR‐II Fluorescence in the Solid State

The commercial surgical sutures (Johnson & Johnson, Ethicon VCP738D), which are widely used in clinic, were discovered to emit strong NIR‐II fluorescence (exposure time: 200 ms), and the intensities decreased with increasing filter length (Figure S11a,b, Supporting Information). These purple sutures are made of Polyglactin 910 and Triclosan that can be degradable, as well as coated with BV‐4B powder and other antibacterial material. Comparatively, the sutures with the same overall components but no BV‐4B did not show fluorescence signals (Figure S12a, Supporting Information). Notably, the plot profiling of the fluorescence accurately calculated the diameter of sutures (Figure S12b–d, Supporting Information).

**Figure 4:**
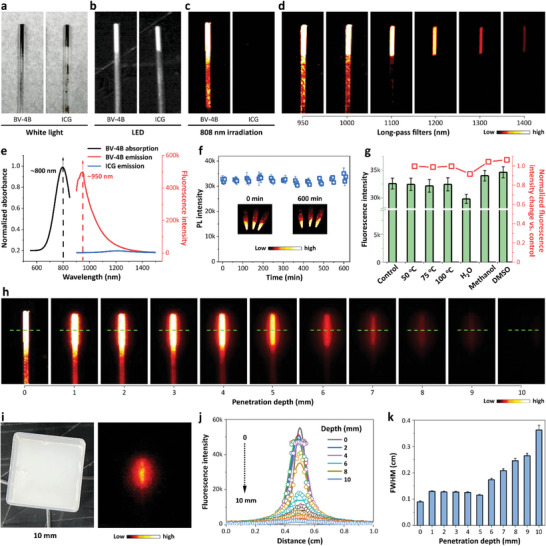
Optical properties of solid BV‐4B in NIR‐II region. a) White‐light, b) LED, and c) NIR‐II imaging on BV‐4B and ICG powder under 808 nm laser excitation (long‐pass (LP) filter: 1000 nm; exposure time: 500 ms). d) NIR‐II imaging of BV‐4B and ICG powder with different LP filters (exposure time: 500 ms). e) Normalized absorbance spectra and PL spectra of BV‐4B and ICG (excitation wavelength: 808 nm) in the solid state. f) The high photostability of BV‐4B exposed to continuous 808 nm laser irradiation for up to 10 h. Inset: fluorescence photographs of BV‐4B powder before (0 h) and after (10 h) irradiation (LP filter: 1000 nm; exposure time: 500 ms). g) No change of fluorescence intensities after heated (50, 75, and 100 °C for 30 min) or dissolved in different solvents (H_2_O, Methanol and DMSO) and then freeze‐dried back to the solid state. All the results were presented as the mean ± s.d from *n* = 5 independent experiments. h) Fluorescence images of capillaries filled with BV‐4B powder, immersed in 1% intralipid with varying depth (0–10 mm). The signals were collected in NIR‐II region under 808 nm laser excitation with 1000 nm filter (exposure time: 200 ms). i) White‐light and fluorescence images of capillary underneath 10 mm of 1% intralipid. j) Decreased fluorescence intensities accompanied with increasing depth. k) The full width at half maxima (FWHM) of fluorescence signals revealing the actual capillary diameter (≈0.1 cm) within 5 mm depth covered.

The solid‐state BV‐4B emitted NIR‐II fluorescence under 808 nm laser irradiation, and it was even imaged with 1300 nm long‐pass (LP) filter. In contrast, indocyanine green (ICG) powder, an FDA approved dye with NIR‐II emission in H_2_O, could not be fluorescently imaged in the solid state because of its well‐studied aggregation‐caused quenching (ACQ) feature (**Figure** **4**a–d). The fluorescence absorbance and emission spectrum showed that BV‐4B in the solid state had the absorbance peak at roughly 800 nm, with 200 nm redshift compared to that in organic solvents. The emission intensity was strong under 808 nm laser irradiation, showing a maximum emission peak at ≈950 nm. The emission tail was extended to over 1300 nm. In contrast, the emission intensity of ICG powder was extremely low (Figure 4e). Under 808 nm laser irradiation, BV‐4B in the solid state showed excellent photostability with similar fluorescence intensity for up to 10 h (Figure 4f). Moreover, the thermostability of BV‐4B was found to be high as evidenced by the constant NIR‐II fluorescence intensity of BV‐4B powder under different temperatures for 30 min. The fluorescence intensity of BV‐4B was also similar after dissolved in H_2_O, methanol and DMSO and then freeze‐dried back to the solid state (Figure 4g; Figure S13, Supporting Information). The absolute quantum yield of BV‐4B under 808 nm irradiation was (0.0312±0.0012)%, similar to several typical organic NIR‐II dyes published previously. These stable optical properties with high fluorescence intensity enabled BV‐4B to become an attractive clinical NIR‐II solid‐state fluorophore capable of being used in clinical conditions.

We further studied whether the NIR‐II fluorescence of BV‐4B had superior advantage in deep imaging over NIR‐I fluorescence. Fluorescence of capillaries filled with BV‐4B powder can be imaged, immersed in even 10 mm of 1% intralipid (Figure 4h–k). The surgical sutures were discriminated underneath 5 mm of intralipid (Figure S14, Supporting Information). However, the fluorescence was not visualized underneath 2 mm of soft tissue in the conventional NIR‐I imaging (Figure S15, Supporting Information). The customized BV‐4B‐coated silicone catheters also showed that the BV‐4B in the solid state was visualized clearly in deep chicken breast tissue because of the strong fluorescence emitting in the NIR‐II region. Three phantoms were still capable of being clearly differentiated covered with 6 mm thickness of soft tissue (Figure S16, Supporting Information), that demonstrated high optical resolution and penetration depth of the NIR‐II imaging for BV‐4B in the solid state. The fluorescence intensity and signal‐to‐background ratio (SBR) decreased with increasing depth of the catheters, but the SBR still reached 2.11 when 5 mm of soft tissue was covered (Figure S17, Supporting Information).

### 2.4 Noninvasive Visualization of Suture Degradation and Catheter Wear

**Figure 5:**
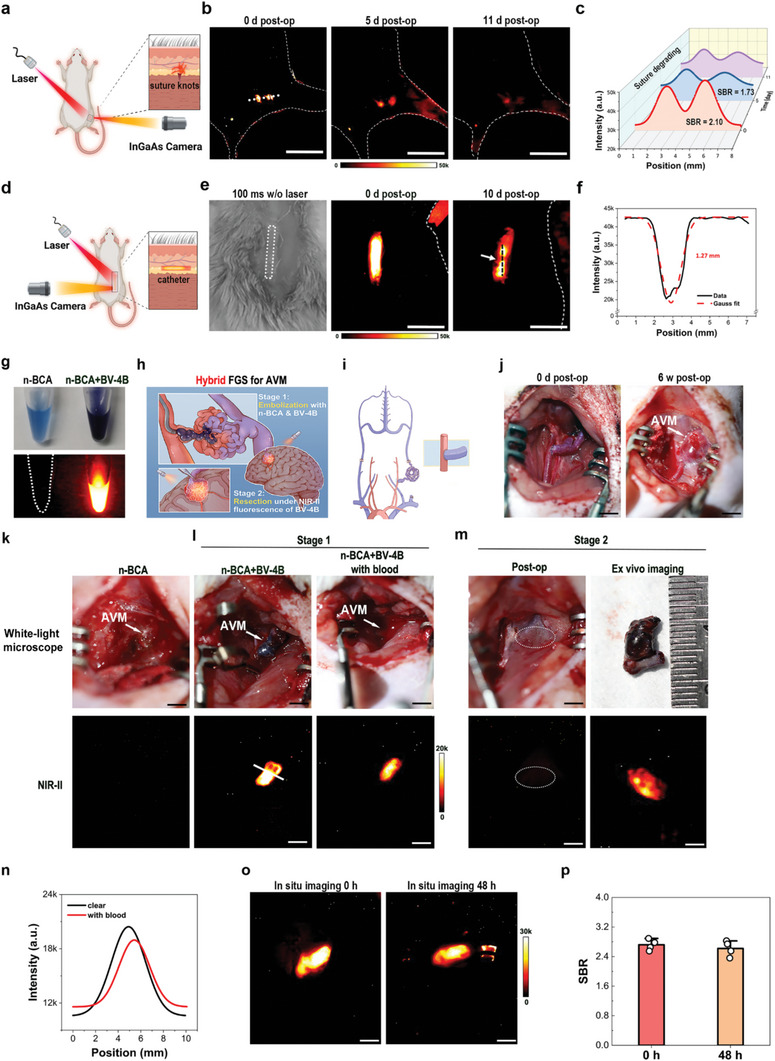
In vivo fluorescence imaging of the surgical sutures and silicone catheters coated with BV‐4B and hybrid fluorescence‐guided surgery of arteriovenous malformation (AVM) under the guidance of n‐Butyl cyanoacrylate (n‐BCA) embolization with BV‐4B. a,b) Fluorescence imaging of the two surgical suture knots in the muscle layer of mice thigh with the intact skin immediately, at 5 d and 11 d, respectively, after operation. (excitation wavelength: 808 nm; emission wavelength: 1000 nm; exposure time: 3000 ms). The dashed line in (b) corresponded to the location of cross‐sectional fluorescence intensity profiles for NIR‐II. c) The quantitative analysis of NIR‐II fluorescence and the decreased mean SBR accompanying with the suture degradation. d,e) The NIR‐II fluorescence of the customized BV‐4B‐coated silicone catheter implanted beneath the tight skin (excitation wavelength: 808 nm; emission wavelength: 1000 nm; exposure time: 1000 ms). The catheter disconnection underneath soft tissue and skin exhibited obvious fluorescence interruption. f) The fluorescence intensity profiling on dashed line in (e) with the full width at half maximum (fwhm) by Gauss fit analysis. g) The photograph of n‐BCA glue without (left) and with BV‐4B (right) (10 mg mL^−1^) showing BV‐4B emitted NIR‐II fluorescence (1000 nm long‐pass filter, exposure time: 1000 ms). h) Illustration of the hybrid operation for brain AVM including the embolization with glue (first stage), e.g., n‐BCA, via the artery catheter and the resection of brain arteriovenous malformation (AVM) nidus several days after embolization (second stage). i) The bypass surgery diagram of the common carotid artery (CCA) and external jugular vein (EJV) to form AVM. j) The AVM formed six weeks after bypass of CCA and EJV. k) No NIR‐II fluorescence from AVM embolization with n‐BCA. l) Strong fluorescence from the AVM after embolization with n‐BCA and BV‐4B (10 mg mL^−1^, 1000 nm LP filter, exposure time: 2000 ms), even hidden under blood. m) No fluorescence signal left in the surgical cavity after resection of the nidus. Strong NIR‐II fluorescence from ex vivo AVM nidus after resected. n) Corresponding cross‐sectional fluorescence intensity profiles along the line in l after Gaussian fitting, easily differentiated fluorescence, even under blood covering. o) 48 h after embolization with n‐BCA and BV‐4B, still strong fluorescence from the in vivo AVM nidus. p) No statistically decreased intraoperative fluorescence SBR at 48 h after embolization surgery (2.72 ± 0.14 vs 2.62 ± 0.21, *p* > 0.05, *n* = 4 biologically independent rats). The bars represented mean ± s.d. SBR: signal‐to‐background ratio. Scale bar: 10 mm.

We further investigated whether the NIR‐II imaging of solid‐state BV‐4B can noninvasively monitor the biodegradation process of medical materials in vivo. With the help of NIR‐II imaging, the surgical suture knots in the muscle layer with intact skin covering was clearly discriminated after operation (**Figure** **5**a). The fluorescence signal gradually attenuated after 5 days (Figure 5b). The average SBR values dropped from 2.10 after operation to 1.73 at 5 days and 1.62 at 11 days (Figure 5c), representing the dynamic degradation process of the BV‐4B‐coated surgical sutures. Interestingly, the dynamic degradation showed a different phenomenon that the fluorescence gradually blurred in another mouse (Figure S18a–c, Supporting Information). The plot profiling indicated that it could not discriminate the previous two fluorescence peaks at 11 days post‐operation (Figure S18d, Supporting Information).

To elucidate potential roles of BV‐4B in monitoring the position and integrity of subcutaneous catheters noninvasively, the silicone catheters coated with BV‐4B and the commercial surgical sutures were used to simulate in vivo applications. BV‐4B can be coated on silicone catheter with great stability *ex vivo* (Figure S19, Supporting Information). Through intact skin and soft tissue, the BV‐4B‐coated silicone catheter was clearly fluorescently imaged (Figure 5d). Furthermore, one millimeter wide of sharp cut was performed to imitate the catheter wear. NIR‐II imaging obviously showed obvious discontinuity of fluorescence, suggesting the catheter disconnected with intact skin (Figure 5e). The plot profiling demonstrated the apparent gap due to the fluorescence signal disconnection, with the SBR of roughly 2.30 achieved (Figure 5f). The fluorescence of even only single strand of 3‐0 suture (diameter 0.20–0.25 mm) was clearly discriminated from normal tissue with intact skin covering (Figure S20a, Supporting Information). The fluorescence intensity gradually decreased after 5 days (Figure S20b, Supporting Information), also demonstrating dynamic suture degradation (Figure S20c, Supporting Information).

### 2.5 Hybrid Fluorescence‐Guided Surgery (Hybrid FGS) of Arteriovenous Malformation (AVM)

n‐Butyl cyanoacrylate (n‐BCA) was one of the most used liquid embolic materials in embolotherapy for vascular malformation.^[^ ^10^ ^]^ Because the viscosity liquid can support an environment for aggregation, BV‐4B emitted strong NIR‐II fluorescence when suspended in the n‐BCA liquids (10 mg mL^−1^ shown in Figure 5g, 1000 ms). This characteristic was highly stable for 48 h (Figure S21, Supporting Information) and the photostability of the complexes including BV‐4B and n‐BCA was excellent after exposure to 808 nm laser irradiation for 30 h (Figure S22, Supporting Information). The suspension of BV‐4B and n‐BCA can achieve a novel surgery strategy (i.e., hybrid FGS) for brain AVM, which comprises two steps: first, embolizing with n‐BCA to decrease blood blow, and bringing BV‐4B into the AVM nidus at the same time; second, resecting via craniotomy with the fluorescence guidance of BV‐4B (Figure 5h). The rat model of AVM was constructed via vascular bypass surgery (Figure 5i; Movie S1, Supporting Information) and was successfully used to simulate AVM after 6 weeks (Figure 5j). n‐BCA was injected into the AVM nidus and demonstrated no fluorescence (Figure 5k). If mixed with BV‐4B, the embolization nidus after n‐BCA polymerization upon contact with blood emitted strong NIR‐II fluorescence, which consecutively guided the whole process of resection even hidden in blood (Figure 5l–n). These two steps were defined hybrid FGS for AVM, which can be used for precise surgery to minimize blood loss and the risk of damaging surrounding normal structures. The interval between embolization and resection was flexible from the finding that 48 h after embolization with n‐BCA and BV‐4B, the AVM nidus in vivo still emitted similar strong fluorescence without significantly changed SBR (2.72 ± 0.14 vs 2.62 ± 0.21, *p* > 0.05, *n* = 4) (Figure 5o,p). Microscopic examination of the resected tissue showed pathologically typical AVM nidus with fluorescence of BV‐4B inside (Figure S23, Supporting Information). Importantly, after adding lipiodol (ethiodized oil, a radio‐opaque contrast agent for radiological intervention treatment) into the suspension (n‐BCA 25% and 50%), the fluorescence of the complex was not affected (Figure S24, Supporting Information).

Most importantly, the surgical sutures coated with BV‐4B are routinely used in clinic after approval by FDA, demonstrating high biosafety of BV‐4B. The histological analysis demonstrated there were no abnormal inflammatory cells infiltration in epidermis, dermis and muscles adjacent to the catheters in the rats with implanted BV‐4B‐coated silicone catheters (Figure S25a–c, Supporting Information). The main secretory organs, including liver, spleen and kidney showed no pathologic change one month after subcutaneous catheter implantation (Figure S25d–f, Supporting Information). There was no uptake of BV‐4B and inflammation response in main organs in the AVM rat models (Figure S26, Supporting Information). All these experiments indicated excellent biocompatibility of BV‐4B.

### 2.6 Clinical Translational Study

**Figure 6:**
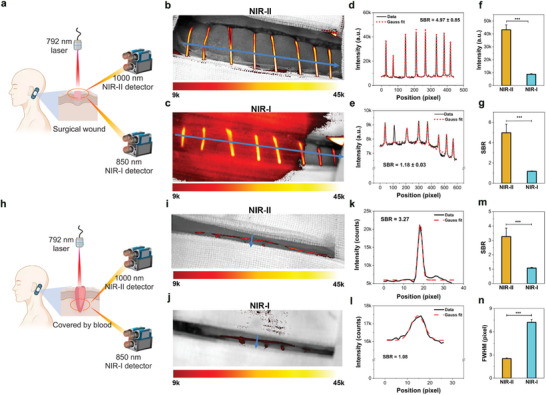
Superior resolution of NIR‐II fluorescence imaging of the commercial surgical sutures in patients intraoperatively. a) Schematic illustration of imaging surgical sutures coated with BV‐4B in surgical wound by multispectral imaging system with the detection of NIR‐II and NIR‐I fluorescence intraoperatively (excitation wavelength: 792 nm; emission wavelength 1000 nm for NIR‐II; emission wavelength 850 nm for NIR‐I). b) The NIR‐II fluorescence imaging of surgical sutures (exposure time: 1000 ms). c) The NIR‐I imaging of the same field with b) (1000 ms). d,e) The cross‐sectional intensity profiles (black) and Gauss fitting (red) of surgical sutures along the blue arrows in the b) NIR‐II and c) NIR‐I images, respectively. f) The intensities of surgical sutures in surgical wound in NIR‐II compared with NIR‐I. g) The mean SBR of surgical sutures in NIR‐II significantly larger than NIR‐I (4.97 ± 0.85, vs 1.18 ± 0.03, *n* = 24 (8 suture strands *3 patients), *p* < 0.01). h) Schematic illustration of imaging surgical sutures in surgical wound and covered by blood by multispectral imaging system with NIR‐II and NIR‐I fluorescence intraoperatively (same parameters with (a)). i) The NIR‐II fluorescence imaging of surgical suture strand covered by blood (exposure time: 1000 ms). j) The NIR‐I imaging of the same field with (i) (1000 ms). k,l) The cross‐sectional intensity profiles of surgical suture strand covered by blood along the blue arrows in the i) NIR‐II and j) NIR‐I images, respectively. m) The mean SBR in NIR‐II significantly larger than NIR‐I (3.27 ± 0.59, vs 1.08 ± 0.05, *n* = 3, *p* < 0.01). n) The FWHM of cross‐sectional profiles in k) NIR‐II and l) NIR‐I (2.52 ± 0.10, vs 7.21 ± 0.39, *n* = 3, *p* < 0.01). SBR: signal‐to‐background ratio. FWHM: Full width half maximum. All results presented as the mean ± s.d from *n* = 24 surgical strands (b,c) and three independent patients (i,j) in clinical research.

Due to the FDA‐approved surgical suture, NIR‐II fluorescence of the commercial sutures coated with BV‐4B was further evaluated in the operation for three patients with vestibular schwannoma. The sutures in surgical wound w or w/o covered by blood were fluorescence imaged by multispectral imaging system with the detection of NIR‐II and NIR‐I fluorescence intraoperatively (**Figure** **6**a,h, excitation wavelength: 792 nm; emission wavelength 1000 nm for NIR‐II; emission wavelength 850 nm for NIR‐I).^[^ ^6a^ ^]^

Compared with NIR‐I fluorescence imaging (Figure 6c), the surgical suture strands can be obviously differentiated by the NIR‐II fluorescence (Figure 6b). Quantitative analysis of cross‐sectional intensity profiles demonstrated higher and shaper intensity peak in NIR‐II than NIR‐I (Figure 6d,e). More importantly, the intensities of surgical sutures in NIR‐II were significantly larger than NIR‐I (Figure 6f, *p* < 0.01), yielding much higher SBR (Figure 6g, 4.97 ± 0.85, vs 1.18 ± 0.03, *n* = 24, *p* < 0.01). In the surgical wounds partly hidden by blood (Figure 6h), the fluorescence signal was also more easily detected by NIR‐II fluorescence than NIR‐I (Figure 6i,j). The cross‐sectional intensity profiles of surgical suture strand covered by blood demonstrated the strength of NIR‐II imaging, especially much sharper intensity peak (Figure 6k,l). The mean SBR in NIR‐II was significantly larger than NIR‐I (Figure 6m, 3.27 ± 0.59, vs 1.08 ± 0.05, *n* = 3, *p* < 0.01). The FWHM analysis of the cross‐sectional profiles demonstrated that the width in NIR‐II was significantly smaller than that in NIR‐I (Figure 6n, 2.52 ± 0.10, vs 7.21 ± 0.39, *n* = 3, *p* < 0.01), more closely with the actual diameter of the suture. This showed the potential utilization in correctly visualizing medical devices fluorescently in vivo in real patients.

## 3 Discussion

Different kinds of medical devices have been implanted into the human body with huge market. For permanently‐implanted medical devices, the incidence of device fracture is relatively high. Taking the ventriculoperitoneal shunt operation as an example, patients need implantation with roughly 50 centimeters‐length silicon catheters underneath the skin permanently, and the silicone catheter associated complication is high.^[^ ^2a^ ^]^ There have been a few feasible, easily used, and non‐harmful imaging techniques to monitor the state of the medical devices without radiation. Apart from solid‐state medical devices, the fabrication with embolic material to make them imaginable is important. AVM and other vascular tumors could be treated with first‐stage embolization surgery following with microsurgical resection. The surgical sutures with modified platinum marker on tips could be used for endovascular embolization and are visible under fluoroscopy.^[^ ^11^ ^]^ Therefore, biocompatible materials with capability for crafting or coating medical devices to make them visible noninvasively is important and represents a novel strategy for medical device monitoring.

NIR‐II fluorescence imaging has been developed quickly, because it has numerous advantages over traditional NIR‐I imaging such as higher imaging resolution and deeper tissue penetration depth. In this context, NIR‐II fluorescence holds high potential for imaging of medical devices in depth and in vivo to monitor their status such as integrity and degradation. However, most of the fluorophores have ACQ feature, which hinders them from being used on medical devices. ICG and methylene blue, the two dyes approved by FDA for clinical use, both have inefficient optical properties (neglectable brightness and poor photostability) when they are in the solid state due to the ACQ phenomenon.^[^ ^9^, ^12^ ^]^ Probes with aggregation‐induced NIR‐II fluorescence can overcome the above limitations and show promising biomedical applications. However, till now none of them has been proved for clinical use or even in clinical research. Therefore, there is an urgent need to discover and develop clinical translatable NIR‐II probes for in vivo imaging of medical devices, which will accelerate the progress of both solid‐state fluorescence material and medical device fields.

Lack of clinical approval use of NIR‐II probes has prompted us to search potential dyes from medical devices that are already broadly used in clinic. In this research, we discovered that BV‐4B, the purple coating of surgical sutures, is an intriguing probe with fluorescence in the NIR‐II range after aggregation. More importantly, BV‐4B has been widely used as a dye in commercial surgical sutures or hair dye for decades without concerns of toxicity, rendering it a promise biocompatible and clinic‐translatable biomaterial for future applications. Our study also confirmed the high biosafety of BV‐4B in vivo. Notably, we found the optical properties of BV‐4B to be very stable, which can be useful for fabrication crafting on medical devices, removing traces of potentially toxic solvent, sterilization, etc. Therefore, BV‐4B can be easily fabricated on medical devices via different chemical engineering protocols in future translation path to clinic.

The BV‐4B, as a potential clinical useful NIR‐II solid‐state dye, further motivated us to extend the potential application in solving the above bottlenecks in clinic. First, biodegradable surgical sutures with BV‐4B coating can be continuously and noninvasively monitored in the whole biodegradation process and demonstrated individual difference in vivo. Second, the wear of the subcutaneous implanted catheters can be clearly diagnosed via lighting‐up them with BV‐4B coating. There are several advantages for coating this stable chemical onto medical devices compared to polymeric nanoparticles using hydrophobic counterions and commercial dyes,^[^ ^9^ ^]^ including easy fabrication, standardized product and direct translation to the clinic. Third, because the embolization glue like n‐BCA can provide the unique environment for chemical aggregation in the suspension state, BV‐4B as a NIR‐II solid‐state probe can smartly guide the safe resection of AVM via hybrid FGS, that is the first‐stage embolization with n‐BCA and BV‐4B and then the second‐stage microsurgical resection under fluorescence. The fluorescence has potential roles of improving the accuracy of resecting AVM and lowering the risk of damaging normal brain compared with the traditional hybrid surgery without fluorescence. More broadly, we foresee that BV‐4B can be coated on many devices for identifying lost device intraoperatively or highlighting urethra, etc.^[^ ^9^, ^12^, ^13^ ^]^

This study provides conceptual design for permanently‐implanted BV‐4B‐coated silicone catheter. However, the standard operation procedure for fabrication of BV‐4B onto medical devices has not been developed for clinical use. The second issue is the limited duration on small animal models for BV‐4B‐coated silicone catheter application. The fabrication crafting could be resolved by specialized companies in medical device development and production in the future.

## 4 Experimental Section

### Spectral Characterization and NIR‐I Imaging of BV‐4B Solution

Absorbance spectra of BV‐4B dissolved in different solvents were taken on an Ultraviolet‐Visible NIR Cary 60 spectrometer (Agilent Technologies) with the background corrected. The NIR‐I fluorescence emission spectrum was captured on a Horiba Fluoromax‐4 spectrofluorometer (excitation wavelength: 570 nm). The absorbance and emission spectrum of different chemicals in the solid state were measured on Horiba FluoroLog‐3 Fluorimeter. NIR‐I imaging of BV‐4B in different solutions were captured by IVIS Lumina II Small Animal Imaging System (Excitation/ Emission: 570 nm/Cy5.5 channel).

### NIR‐II Imaging and Stability of BV‐4B Powder

The NIR‐II imaging of BV‐4B and ICG in the solid state were performed on a 2D InGaAs array NIR‐II system (Princeton Instruments). The excitation was provided by an 808 nm diode laser through an optical fiber and collimator. Fluorescence emission was collected with 1000, 1100, and 1300 nm long‐pass (LP) filters (Thorlabs). BV‐4B powder was heated to 50, 75, and 100 °C for 30 min, respectively, and it was then cooled to room temperature for NIR‐II imaging. Besides heating, BV‐4B powder was also dissolved in H_2_O, methanol and DMSO respectively, and then freeze‐dried to powder. All powder after the above process, were exposed to 808 nm laser for NIR‐II imaging and a 1000 nm LP filter was used. A continuous laser irradiation up to 10 h was applied for BV‐4B powder, and the NIR‐II imaging was performed every hour after laser irradiation. ImageJ software was used for analyzing the images. The absolute quantum yield of BV‐4B powder was measured in the integrating sphere (QuantaPhi‐2, HORIBA Scientific, Canada). The calculation range of the emission spectrum was 885–1180 nm (Fluorolog‐QM, HORIBA Scientific, Canada). In addition, the excitation energy was strong during the test, and the attenuation film was used for the test of the four‐curve method, and finally the correction factor (0.009) was used for conversion.

### Density Function Theory Calculation

Gaussian 09 software was used to perform the theoretic calculations. Ground state optimization was carried out using opt B3LYP/6‐31G(d) method, and from which the energy levels of frontier orbitals were obtained.

### Penetration Depth of NIR‐II Imaging

FDA Food Grade Silicone catheters with translucent color were purchased from the Cold & Colder (United States). The absorbable 3‐0 coated VICRYL Plus surgical sutures, catalog number VCP738D, were obtained from Ethicon company (Johnson & Johnson Medical). The surgical knots and silicone catheter coated by BV‐4B were prepared for further application studies in vivo. Chicken breast was used to mimic the penetration depth. Different thickness of chicken breasts (1, 2, 3, 4, 5, 6, and 7 mm) were covered on surgical suture knots and silicone catheter for NIR‐II imaging. An 808 nm laser and a 1000 nm LP filter were used.

### Preparation of BV‐4B‐Coated Silicone Catheter and Stability Test

The commercially available PDMS consisted of pre‐polymer (Sylgard 184) and curing agent, which mixed together in definite proportions to get PDMS. First, 20 mg of pre‐polymer (Sylgard 184) was mixed with 2 mg of curing agent with a spatula. The mixture was fully mixed until the formation of a homogeneous and transparent solution to get PDMS. BV‐4B was dissolved in methanol and diluted with THF to a concentration of 1 mmol L^−1^ for a total volume of 500 µL. The PDMS was then added to the BV‐4B solution and stirred for several minutes until fully mixed. Then the tiny bubbles in this mixture was removed using a vacuum. After that, silicone catheter was put into the mixture for 2 min and then removed. The film on the silicone catheter was left to dry at room temperature for 4 days. In order to detect the stability of BV‐4B on the catheter, the BV‐4B‐coated dry silicone catheter was soaked in PBS solution for 7 days. On the following 1st/3rd/7th day, the color of PBS solution was observed, and the absorption spectrum was studied.

### Animal Experiment

All animal experiments were approved by the Administrative Panel on Laboratory Animal Care at Beijing Neurosurgical Institute (202 304 006). Seven‐week‐old female BALB/C mice were from the Charles River for all in vivo imaging. Before operations, the hair of the hind leg and back was removed (*n* = 3 in respectively lymphatic imaging, suture knots and silicone catheter implantation). All mice and rats were anesthetized with 2 L min^−1^ O_2_ gas mixed with 3% isoflurane. For lymphatic imaging, the BV‐4B dye (200 µM, 50 µL) was injected subcutaneously into the footpad in the prone position. The mice were imaged in the IVIS Lumina II Small Animal Imaging System (Excitation wavelength: 570 nm; Emission wavelength: Cy5.5 channel) after 30 min, with the local skin on the hind leg removed. For surgical suture knots imaging, a 1.0 cm‐length skin incision was made on the hind leg, and then two to three suture knots were performed in the muscle layer, and lastly the skin was closed using nonabsorbable surgical sutures. The mice were transferred to an imaging stage beneath the laser and NIR‐II small animal imaging setup (808 nm excitation at a power density of 140 mW cm^−2^, 1000 nm LP filter, exposure time: 3000 ms).^[^ ^14^ ^]^ The NIR‐II imaging was repeated respectively at 5, 11 days with the same condition. For silicone catheters coated with BV‐4B, two 3 mm‐length skin incisions roughly 1 cm apart were performed, then hemostatic forceps were passed through from one incision to the other incision underneath intact skin. The silicone catheter was pulled underneath skin with hemostatic forceps, and then it was fixed onto muscle before closing the skin incision. The second operation was performed to cut the catheter in the middle with micro scissors after 10 days with skin closed. The mice were imaged again with similar condition with suture knots imaging (exposure time: 1000 ms). The AVM models were performed in 3‐month‐old female rats. The bypass of the common carotid artery (CCA) and external jugular vein (EJV) on the left side were done with 10‐0 Prolene sutures (Ethicon, Inc., Somerville, NJ) under the surgical microscope.^[^ ^15^ ^]^ After 6 weeks, the ultrasound scanning was confirmed that the diameter of AVM nidus was at least 5 millimeters for further experiments. BV‐4B mixed with n‐BCA (20 mg mL^−1^, 200–500 µL) was injected into the fistula via the external carotid artery. The resection operation was performed under the microscope and fluorescence imaging (*n* = 4 respectively for immediately and 48 h after embolization).

### Clinical Study

The fluorescence imaging of the commercial surgical sutures in operations for brain tumor was registered in Chinese Clinical Trial Registry (ChiCTR2000033430). The NIR‐II instrument was used in the previous clinical experiment.^[^ ^6a^ ^]^ Three patients with vestibular schwannoma performed by retrosigmoid approach gave consent for this clinical study.

### Ex Vivo Analysis of Toxicity of BV‐4B‐Coated Catheter and Embolization

The mice receiving sutures and silicone catheter implantation were sacrificed 30 days after operations. The skins, soft tissue and the muscle around the implanted catheter and all organs were embedded by the optimal cutting temperature (OCT) compound (Tissue‐Tek, Sakura Finetek, USA). The samples were cut into 6 µm sections in cryostat and transferred onto slides for hematoxylin and eosin (H&E) stain with Shandon rapid Chrome kit (Thermo Scientific, USA). Rats with AVM were embolized with n‐BCA and BV‐4B mixture. After 48 h, the AVM nidus were resected under the fluorescence‐guided surgery and then the rats were sacrificed. Slides with organs and the AVM nidus were stained by H&E. Moreover, 2‐(4‐Amidinophenyl)−6‐indolecarbamidine dihydrochloride (DAPI) was also used to stain the AVM nidus, in order to indicate the soft tissue and the embolization materials.

### Statistical Analysis

The photoluminescence measurement was performed to quantitate NIR optical signal intensity through the Image J 1.45x software (National Institutes of Health, Bethesda, MD). Data are given as mean ± s.d (standard deviation). Statistical significance was determined by a two‐tailed Student's t test. A P‐value of <0.05 was considered significant.

## Conflict of Interest

The authors declare no conflict of interest.

## Author Contributions

D.L., H.S., Q.Q., and B.C. contributed equally to this work. D.L., H.S., Q.Q., J.T., Z.H., B.C., W.J., and Z.C. conceived and designed the experiments. D.L. and H.S. performed the fluorescent imaging experiments. H.S., Y.L., B.C., and L.X. performed the chemical experiments and further analysis. K.Q., C.Q., and B.C. did the theoretical calculation. D.L., Y.J., and H.S. performed the mice operations and in vivo fluorescence imaging‐guided surgery. D.L. and S.H. performed other biological experiments. D.L., Z.Z., X.S, Y.Z, Z.L, B.L., L.C., and P.K. conducted the clinical study. N.M., W.C., H.C., and Y.J. took part in the discussion. All authors participated in the interpretation of the reported experiments or results. D.L., H.S., Q.Q., B.C., Z.H., and Z.C. wrote and revised the manuscript. All other authors reviewed the final version of the manuscript.

## Supporting information

Supporting Information

Supplemental Movie 1

## Data Availability

The data that support the findings of this study are available on request from the corresponding author. The data are not publicly available due to privacy or ethical restrictions.
