# Supplementary material for: Clinically Translatable Solid‐State Dye for NIR‐II Imaging of Medical Devices
Source: Adv Sci (Weinh). 2023 Nov 9;10(36):2303491. doi: 10.1002/advs.202303491 (PMC10754084; doi:10.1002/advs.202303491)
Supplement: Supplementary file 1 — Supporting Information [file ADVS-10-2303491-s002.pdf]

ADVANCED SCIENCE
Open Access

Supporting Information

for *Adv. Sci.*, DOI 10.1002/advs.202303491

Clinically Translatable Solid-State Dye for NIR-II Imaging of Medical Devices

Deling Li, Hui Shi, Qingrong Qi, Baisong Chang, Yuanwen Jiang, Kun Qian, Xiudong Guan, Peng Kang, Ning Ma, Yuan Zhang, Zeyu Zhang, Xiaojing Shi, Chunrong Qu, Yilei Wu, Weiyu Chen, Hao Chen, Baowang Li, Liangpeng Chen, Ziyang Li, Shunchang Ma, Lingyun Xu, Yanrong Zhang, Jie Tian, Zhenhua Hu*, Wang Jia* and Zhen Cheng**

Supplementary Materials:

Supplementary Figures:

S1. Photograph of BV-4B in H_2O and different organic solvents.

S2. Photograph and normalized PL spectra of BV-4B in DMSO/H_2O mixtures.

S3. Photograph of BV-4B in THF/Methanol mixtures with different THF fractions (f_{THF}).

S4. The three-dimensional spectroscopy of BV-4B in different solvents: a) in CH_3OH; b) in THF:CH_3OH=1:1; c) in THF:CH_3OH=99:1.

S5. The enhanced PL intensity of BV-4B dissolved in THF with different concentrations after incubation for 24 hours.

S6. BV-4B aggregation demonstrated with the dynamic light scattering analysis.

S7. The dihedral angles between a-b, a-c and b-c of BV-4B in methanol.

S8. (**a**) Photograph and (**b**) NIR-I imaging of BV-4B with different concentration of FBS. (**c**) PL spectra of BV-4B in different concentration of FBS.

S9. NIR-I imaging of BV-4B *in vivo*.

S10. The effect of temperature on the a) emission spectra and b) the maximum emission intensity of BV-4B dye. The effect of time on the c) emission spectra and d) the maximum emission intensity of BV-4B dye.

S11. Fluorescence imaging of BV-4B-coating surgical sutures gridding under 808 nm laser excitation with different long-pass filters (LF).

S12. Analyzing suture diameters *via* NIR-II fluorescence imaging of surgical sutures coating with BV-4B dye.

S13. NIR-II imaging of BV-4B in the solid state after processing in **f** of fig. 4 under 808 nm laser excitation (exposure time: 500 ms).

S14. The penetration and resolution of NIR-II imaging of commercial surgical suture coated with BV-4B.

S15. The penetration and resolution of NIR-I imaging of commercial surgical sutures coated with BV-4B.

S16. The penetration and resolution of NIR-I imaging of customized silicone catheters coated with BV-4B.

S17. The phantom study of the fluorescence the customized silicone catheters coated with BV-4B as a function of depths (0 - 6 mm) under 808 nm excitation.

S18. Different degradation patterns of the BV-4B-coated surgical suture knots.

S19. The absorption spectrum of the PBS solution at the following 1st/3rd/7th day with BV-4B-coated silicone catheter in PBS, compared with the fluorescence intensity background of BV-4B.

S20. *In vivo* fluorescence imaging of the surgical suture underneath the skin in mice.

S21. The NIR-II fluorescence stability of BV-4B in n-BCA with different concentrations.

S22. The NIR-II fluorescence photostability of the complexes including BV-4B and n-BCA exposed to continuous 808 nm laser irradiation for 30 h.

S23. Microscopic examination of the resected tissue from the arteriovenous malformation (AVM) nidus of the rat.

S24. No change of the NIR-II fluorescence from the suspension with n-BCA and BV-4B (30 mM) after adding lipiodol (ethiodized oil), as a radio-opaque contrast agent.

S25. Tissue toxicity analysis based on histologic staining on soft tissue around catheters and main organs.

S26. Tissue toxicity analysis based on histologic staining of the main organs from the rat with arteriovenous malformation (AVM) which receive the hybrid fluorescence guided surgery.

Supplementary Tables:

Supplementary Table 1. The solubility of BV-4B.

Supplementary Table 2. The dihedral angles between a-b, a-c and b-c of BV-4B in different solvents.

Supplementary Video.

Description: The video showing successful bypass of the common carotid artery (CCA) and external jugular vein (EJV) in the rat.

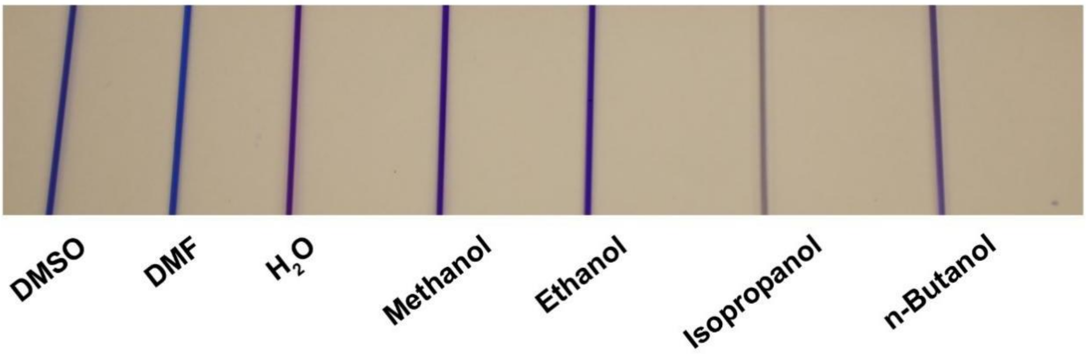

Supplementary Fig. 1 | Photograph of BV-4B in H_2O and different organic solvents.

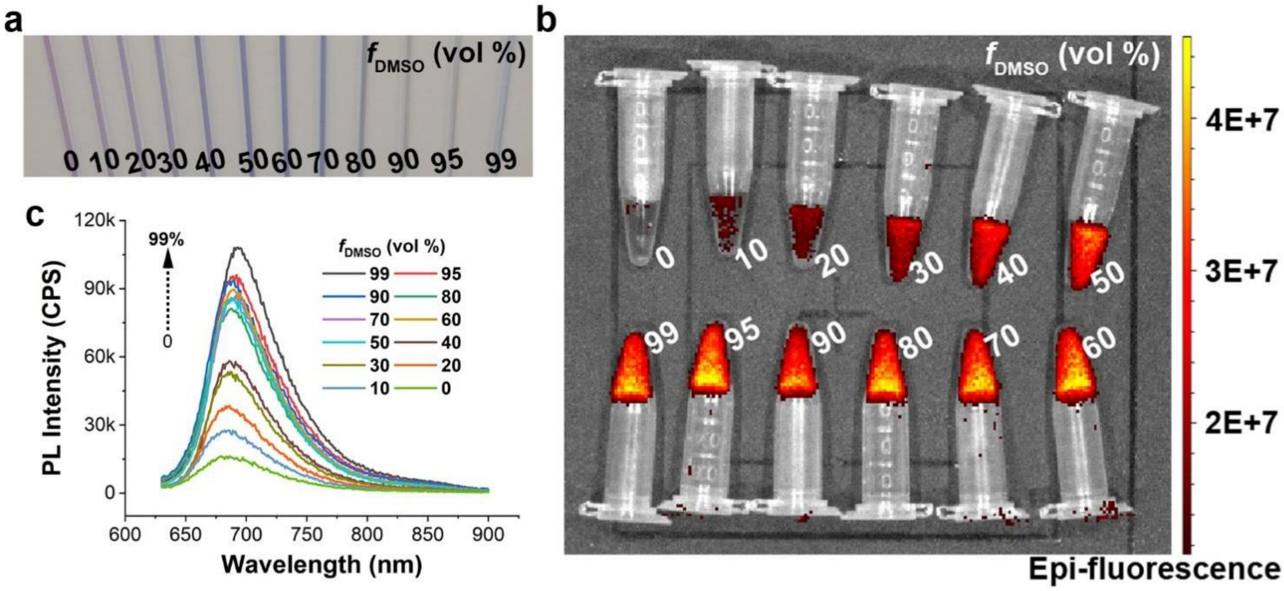

Supplementary Fig. 2 | Photograph and normalized PL spectra of BV-4B in DMSO/H_2O mixtures. a, Photograph of BV-4B in DMSO/H_2O mixtures with different DMSO fractions (f_{DMSO}). **b** and **c,** NIR-I imaging and PL spectra of BV-4B in DMSO/H_2O mixtures with different DMSO fractions (f_{DMSO}).

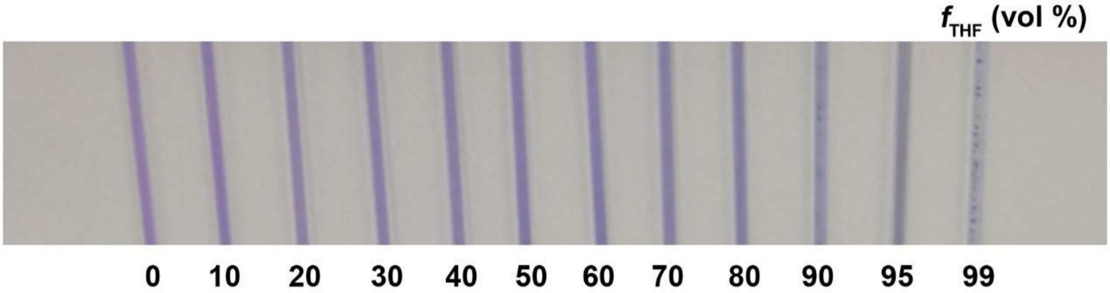

Supplementary Fig. 3 | Photograph of BV-4B in THF/Methanol mixtures with different THF fractions (f_{THF}).

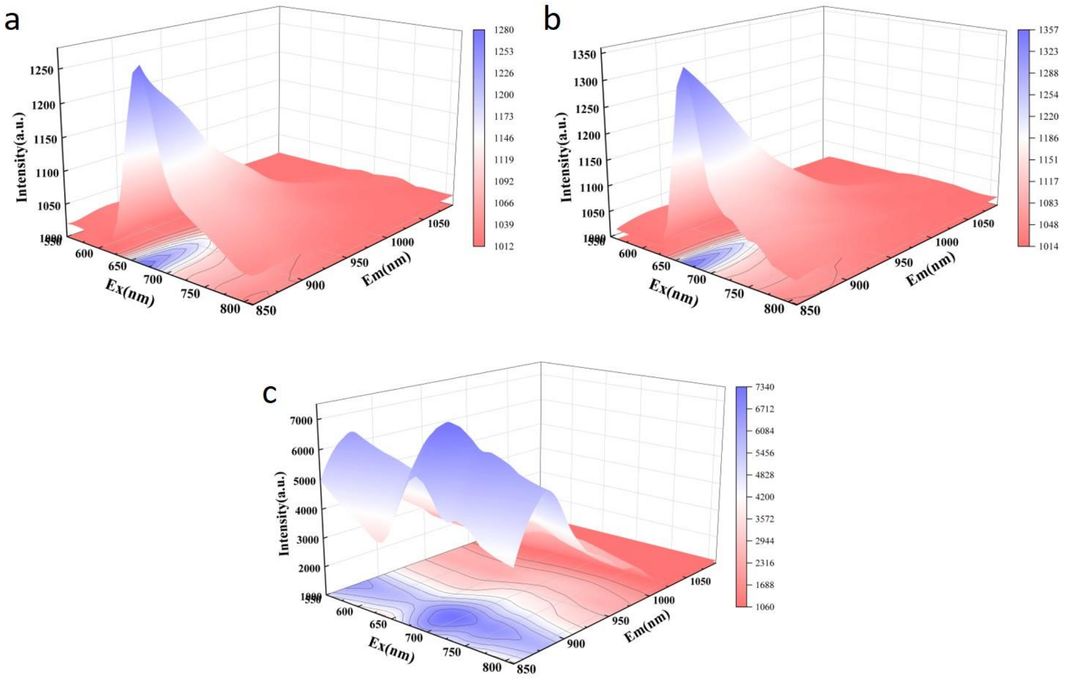

Supplementary Fig. 4 | The three-dimensional spectroscopy of BV-4B in different solvents: a) in CH_3OH; b) in THF:CH_3OH=1:1; c) in THF:CH_3OH=99:1.

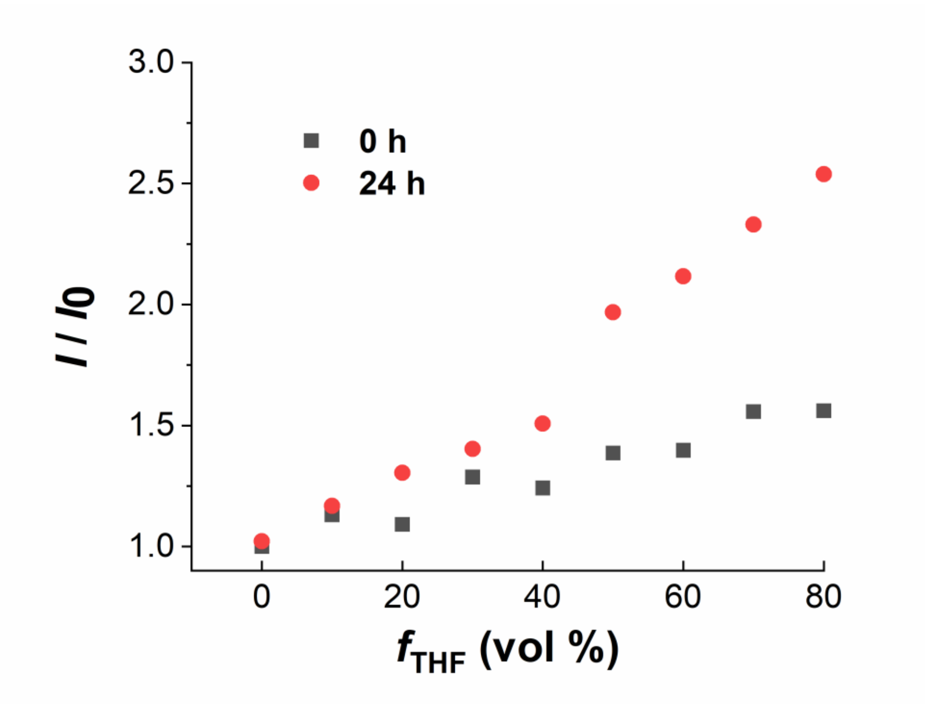

Supplementary Fig. 5 | The enhanced PL intensity of BV-4B dissolved in THF with different concentrations after incubation for 24 hours.

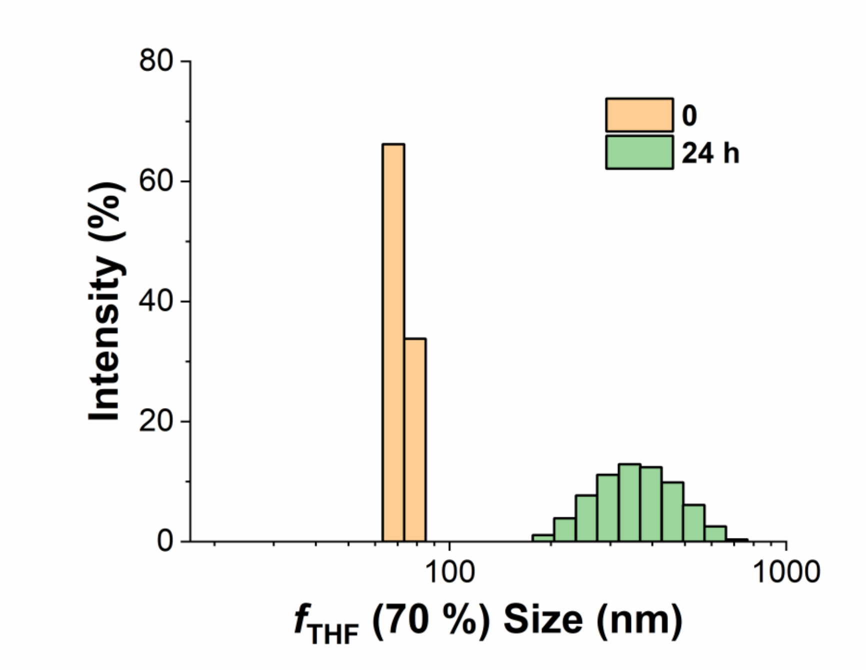

Supplementary Fig. 6 |BV-4B aggregation demonstrated with the dynamic light scattering analysis.

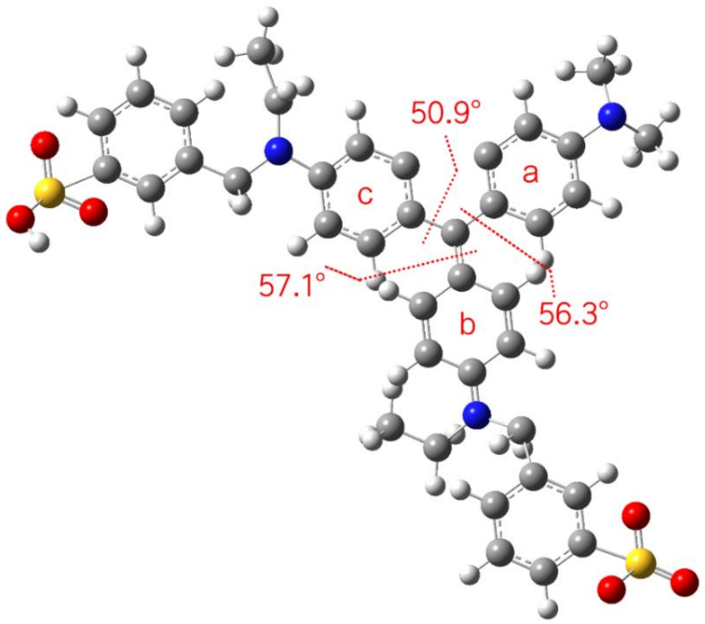

Supplementary Fig. 7 | The dihedral angles between a-b, a-c and b-c of BV-4B in methanol.

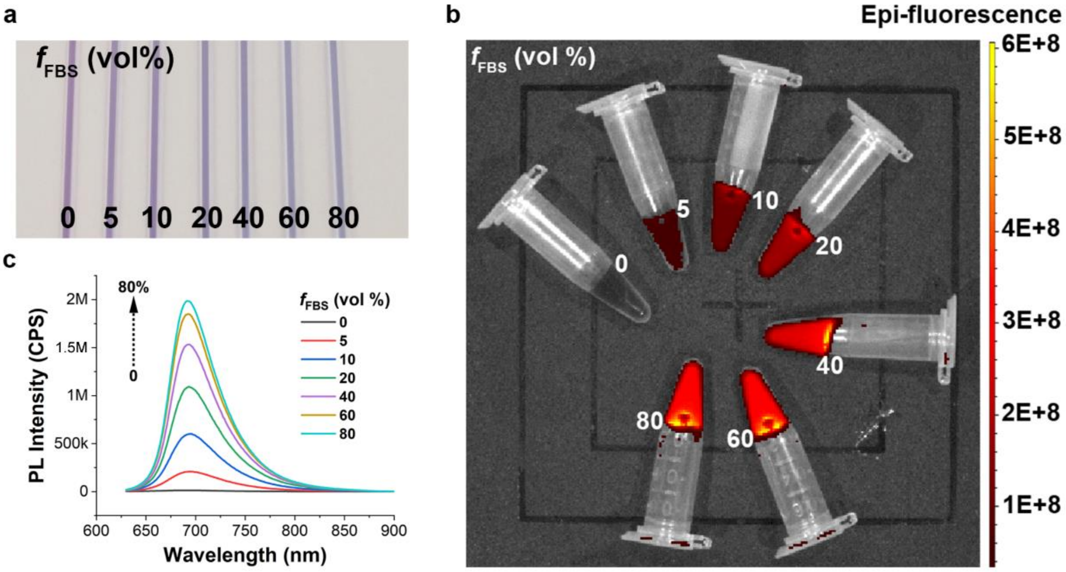

Supplementary Fig. 8 | a, Photograph and **b,** NIR-I imaging of BV-4B with different concentration of FBS. **c,** PL spectra of BV-4B in different concentration of FBS.

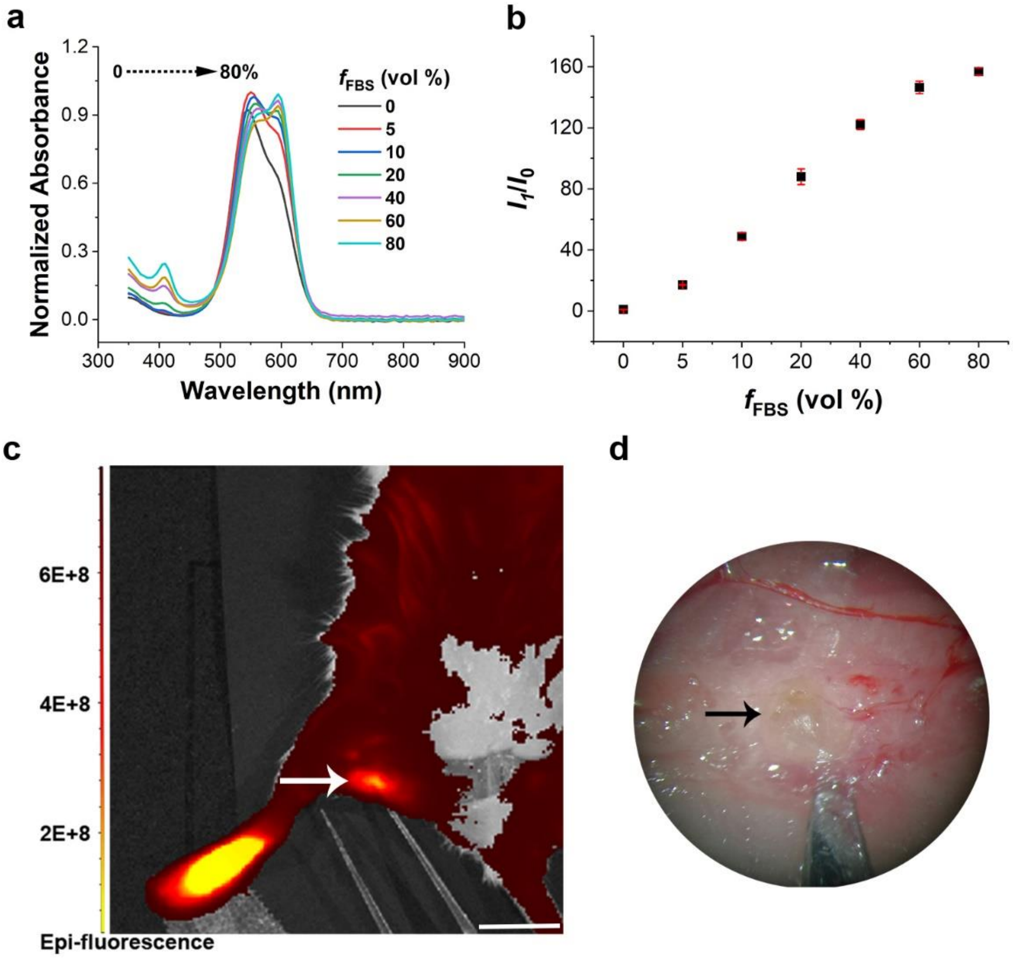

Supplementary Fig. 9 | NIR-I imaging of BV-4B *in vivo*. a, Normalized absorption spectra of BV-4B in PBS with different concentration of FBS. **b,** PL intensity of BV-4B with different concentration of FBS. (excitation wavelength: 570 nm; emission wavelength: 700 nm). **c,** Popliteal lymph node (white arrowheads) identified using NIR-I imaging at 30 min after BV-4B solution in PBS was injected from the footpad in the prone position. **d,** Photograph of the popliteal lymph node under surgical microscope. Scale bar: 10 mm.

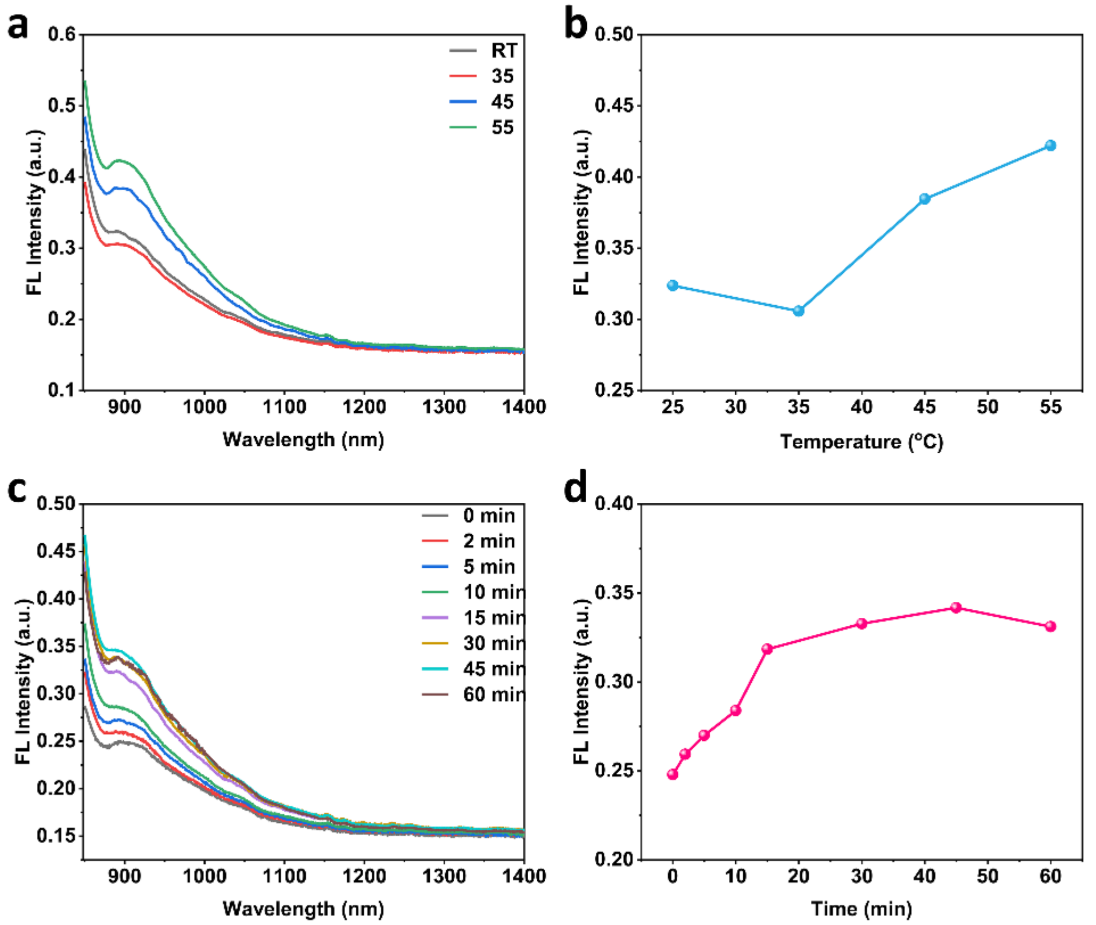

Supplementary Fig. 10 | The effect of temperature on the a) emission spectra and b) the maximum emission intensity of BV-4B dye. The effect of time on the c) emission spectra and d) the maximum emission intensity of BV-4B dye.

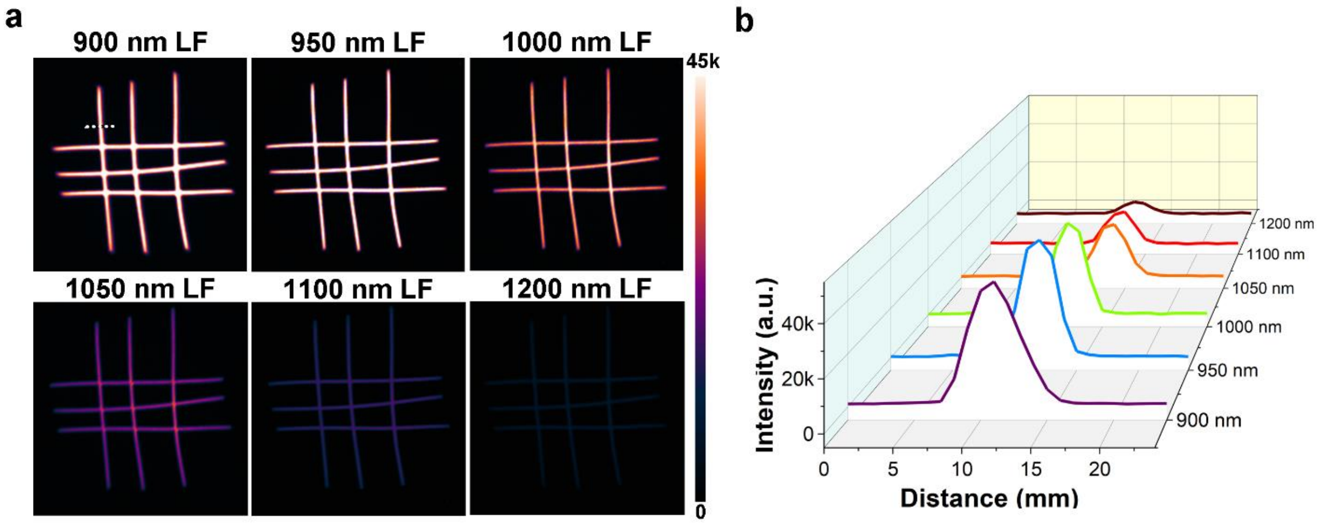

Supplementary Fig. 11 | Fluorescence imaging of BV-4B-coating surgical sutures gridding under 808 nm laser excitation with different long-pass (LP) filters. **a**, Fluorescence of the sutures with different LP filters (900, 950, 1000, 1050, 1100 and 1200 nm) (exposure time: 500 ms). **b**, The decreasing intensities accompanied with increasing LP length by analysis of the fluorescence plot profiling (indicated with white dash line in **a**).

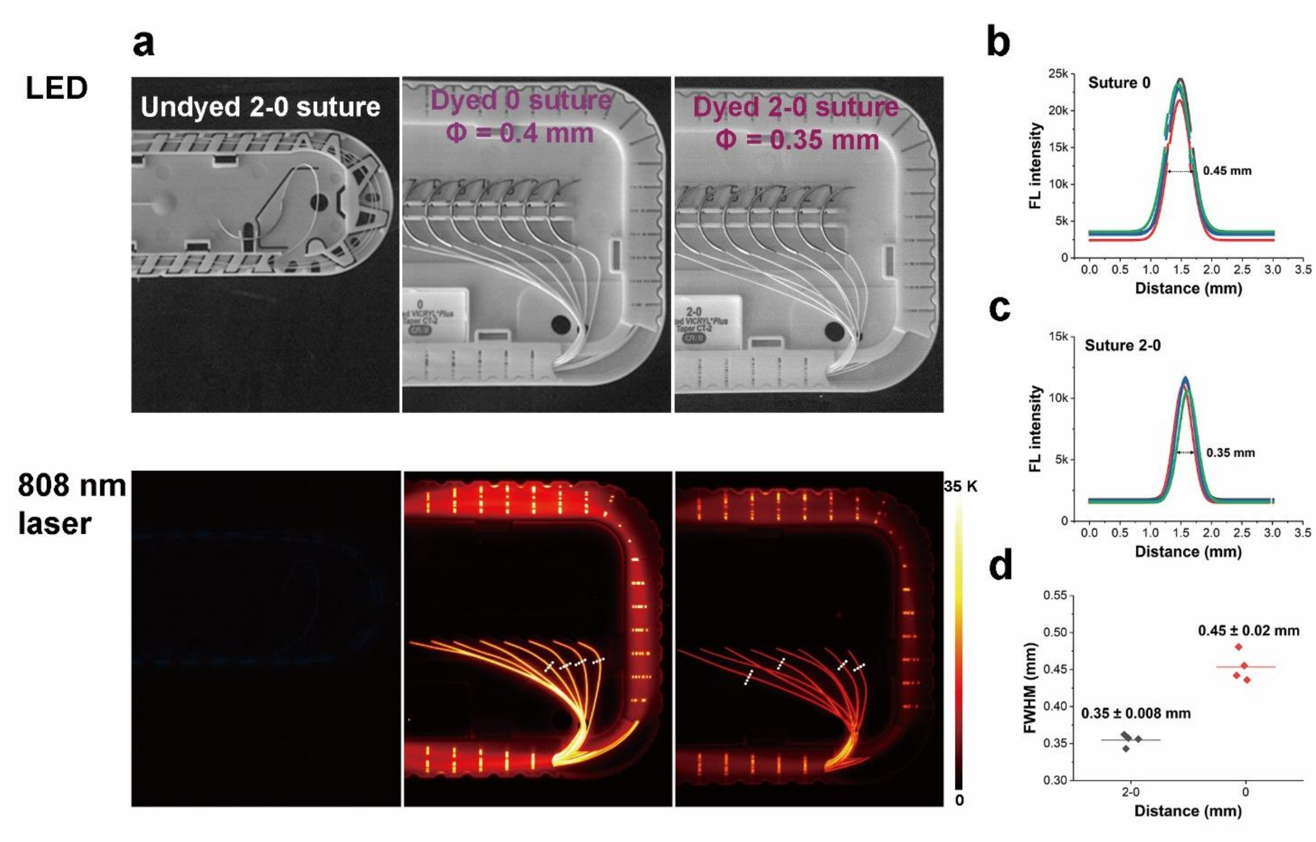

Supplementary Fig. 12 | Analyzing suture diameters *via* NIR-II fluorescence imaging of surgical sutures coating with BV-4B dye. **a,** the LED and fluorescence imaging of surgical sutures with different diameters (suture 0, 0.4 mm; suture 2-0, 0.35 mm), respectively without and with BV-4B coating (exposure time: 200 ms). **b**,**c**, Plot profiling of fluorescence of Suture 0 (**b**) and Suture 2-0 (**c**) (indicated with white dash lines in **a**) and the Full Width at Half Maximum (FWHM). **d**, The mean ± s.d. of FWHM by analyzing four independent transverse section of sutures.

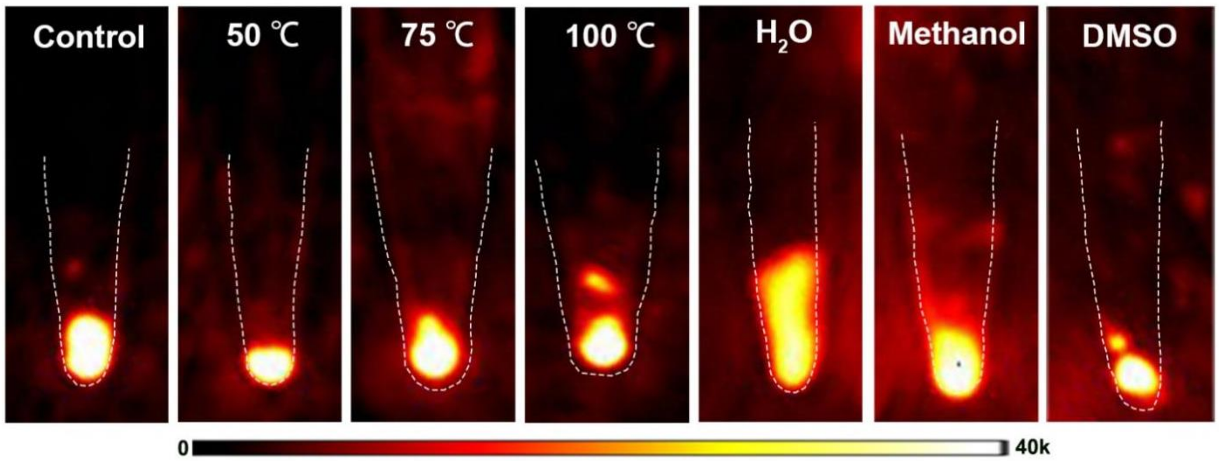

Supplementary Fig. 13 | NIR-II imaging of BV-4B in the solid state after several processing in f of fig. 4 under 808 nm laser excitation (exposure time: 500 ms).

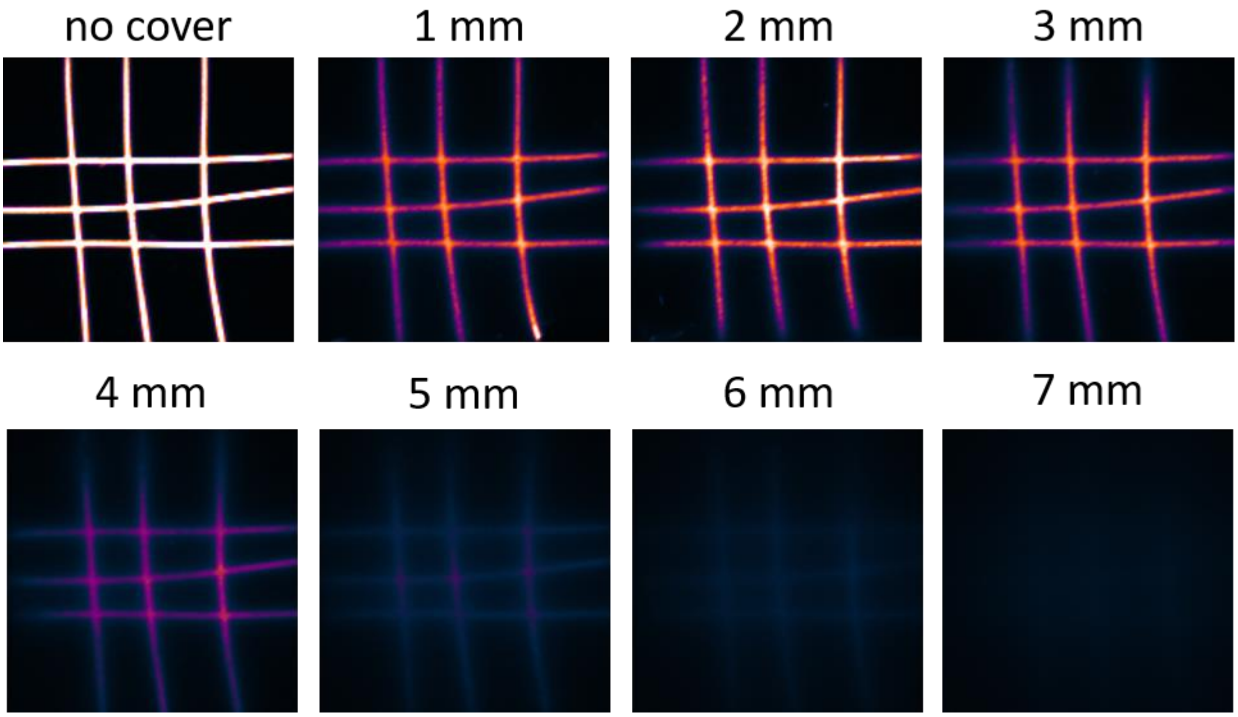

Supplementary Fig. 14 | The penetration and resolution of NIR-II imaging of commercial surgical sutures coated with BV-4B. Surgical 3-0 BV-4B-coated sutures covered by 1% intralipid with various thickness (1, 2, 3, 4, 5, 6, 7 mm). Obvious fluorescence shown under 808 nm laser irradiation (1000 nm long-pass filter, exposure time: 500 ms).

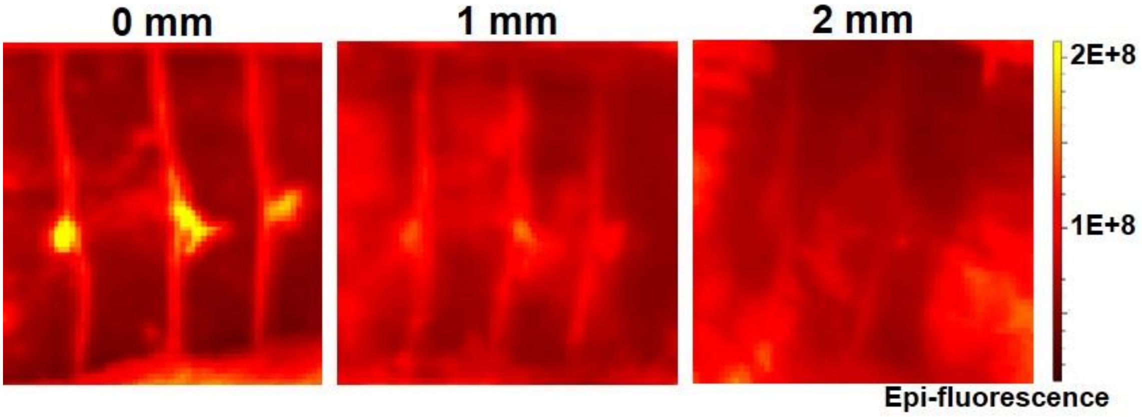

Supplementary Fig. 15 | The penetration and resolution of NIR-I imaging of commercial surgical sutures coated with BV-4B. Three surgical suture knots with 3-0 BV-4B-coated sutures covered by chicken breast tissue with various thickness (0, 1, 2 mm) (excitation wavelength: 570 nm; emission wavelength: Cy5.5 channel; exposure time: 1000 ms).

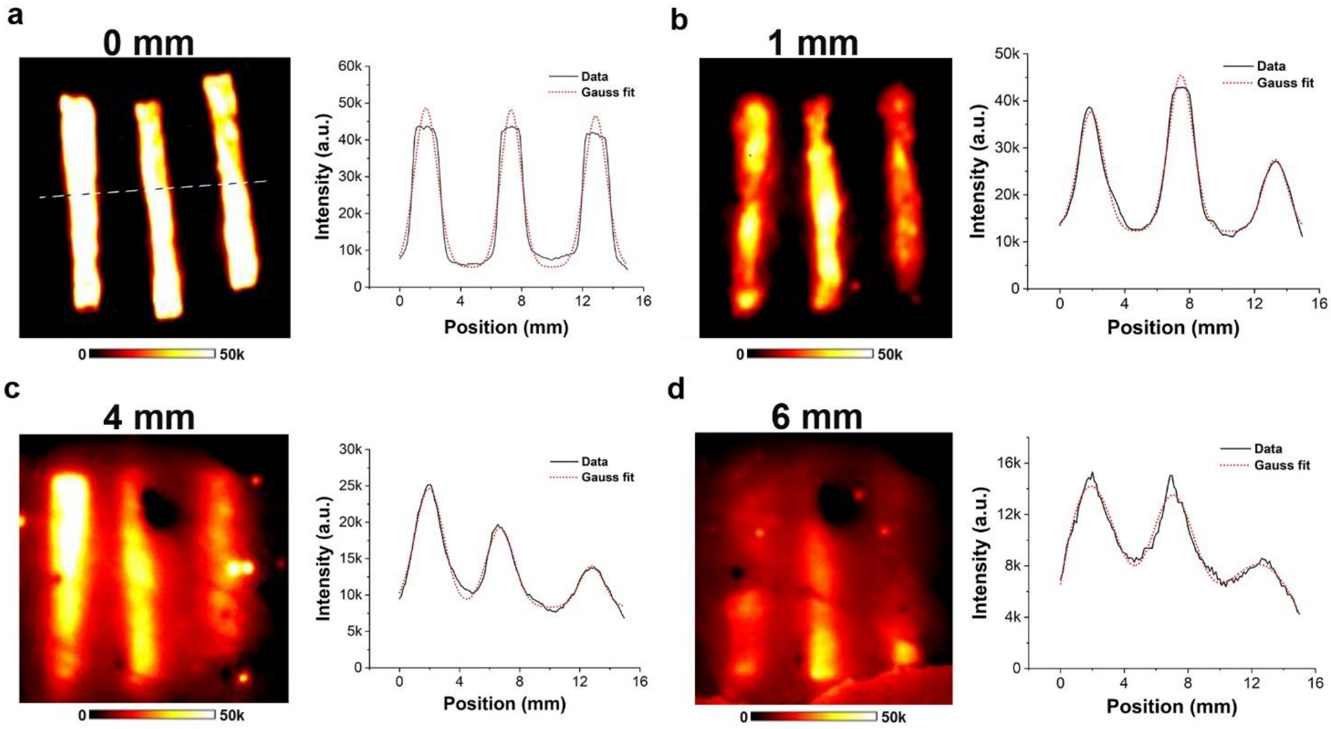

Supplementary Fig. 16 | The penetration and resolution of NIR-I imaging of customized silicone catheters coated with BV-4B. Three customized BV-4B-coated silicone catheters fluorescently imaged underneath chicken breast tissue. Dotted line corresponds to the location of cross-sectional fluorescence intensity profiles. The Gauss fit analysis for further quantitative analysis of the mean intensity and SBR relevant to depth.

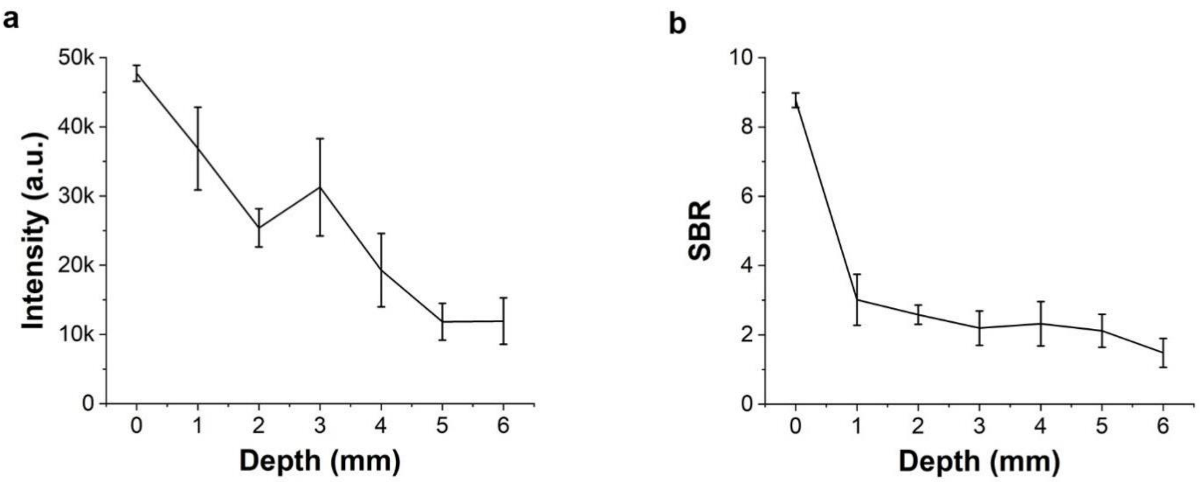

Supplementary Fig. 17 | The phantom study of the fluorescence the customized silicone catheters coated with BV-4B as a function of depths (0 - 6 mm) under 808 nm excitation (0.14 W cm^{-2} power density, 1000 ms exposure time and 1000 nm long-pass optical filter). **a,** The decreased NIR-II fluorescence signal with increasing depth of the customized silicone catheters, as well as the mean SBR in **b**. SBR: signal-to-background ratio. All results presented as the mean ± s.d from n = 5 independent experiments.

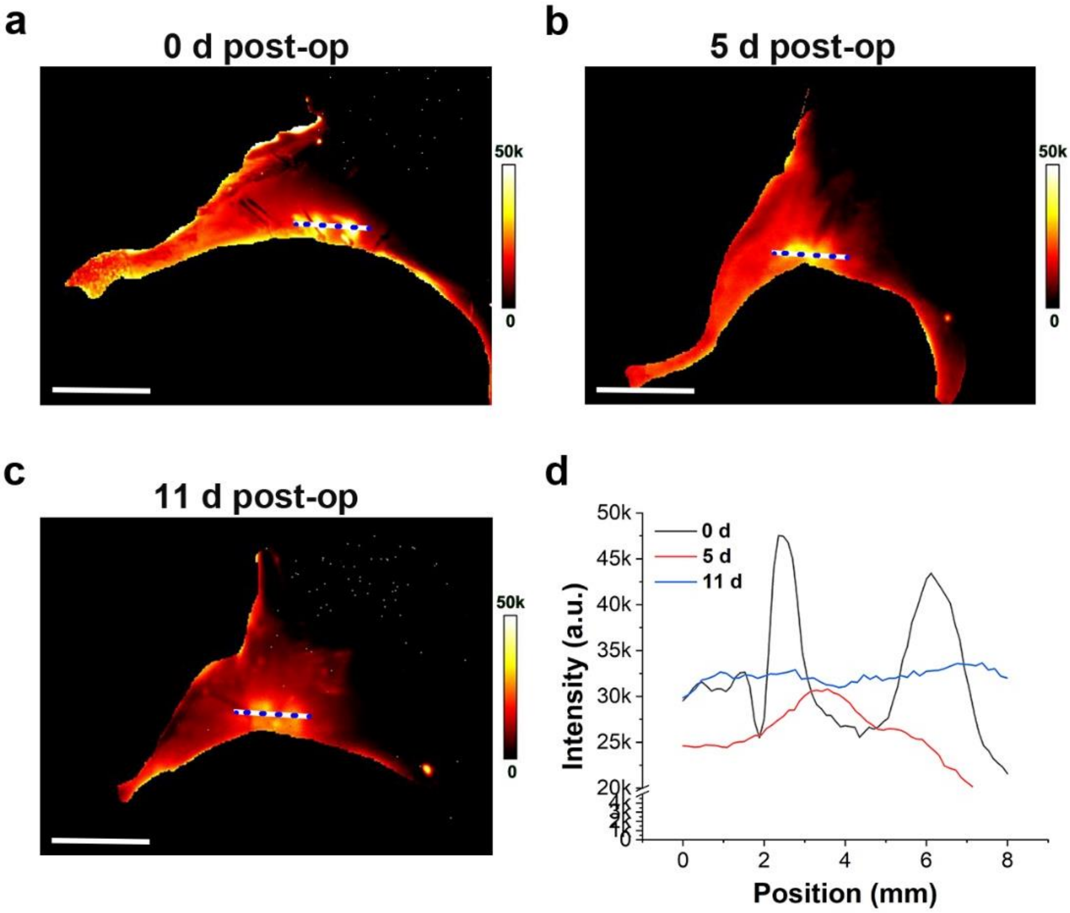

Supplementary Fig. 18 | Different degradation patterns of the BV-4B-coated surgical suture knots. a, Two surgical knots in the muscle layer of mice thigh fluorescently imaged with the intact skin immediately after operation. (excitation wavelength: 808 nm; emission wavelength: 1000 nm; exposure time: 3000 ms). The dashed line corresponds to the location of cross-sectional fluorescence intensity profiles for NIR-II. **b,** The fluorescence began blurred after 5 days; it was difficult to discriminate different knots. **c,** The dramatically dispersed fluorescence within previous area after 11 d. **d,** Plot profiling showed the obvious fluorescence peaks gradually flattened and diminished. Scale bar: 10 mm.

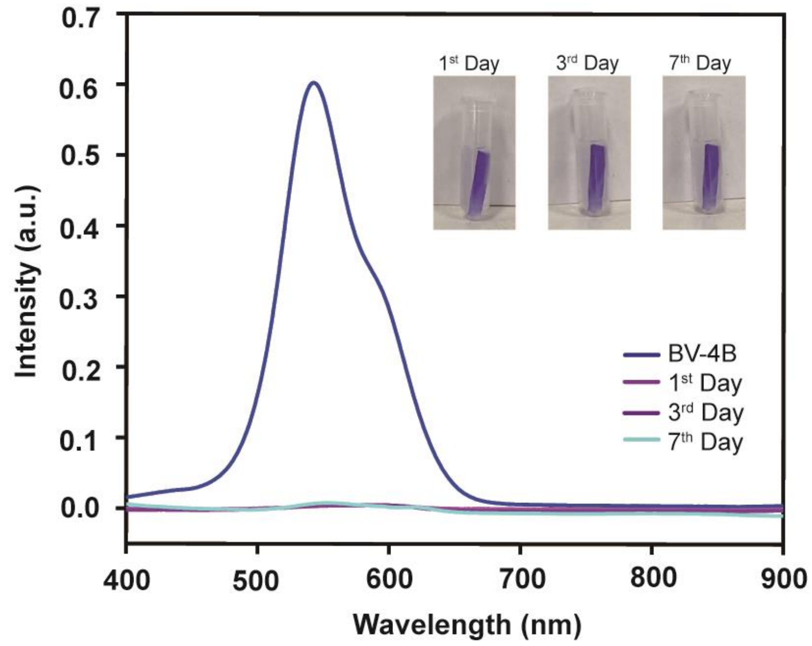

Supplementary Fig. 19 | The absorption spectrum of the PBS solution at the following 1st/3rd/7th day with BV-4B-coated silicone catheter in PBS, compared with the fluorescence intensity background of BV-4B.

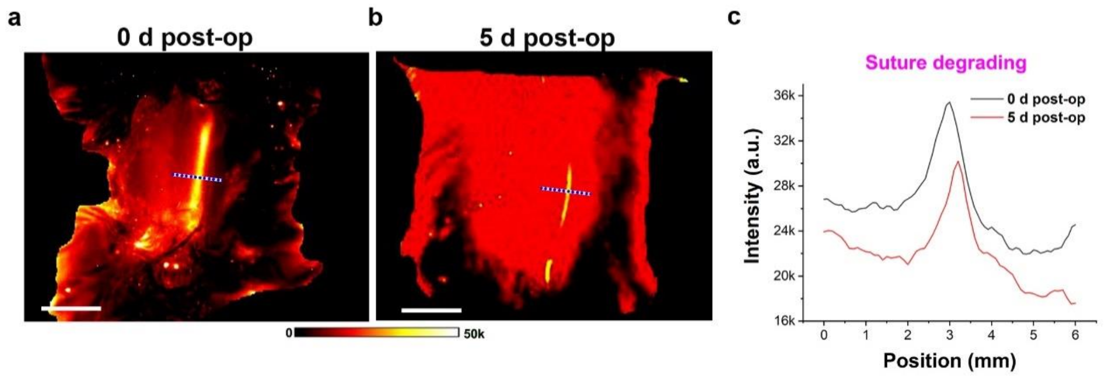

Supplementary Fig. 20 | *In vivo* fluorescence imaging of the surgical suture underneath the skin in mice. a, The fluorescence from a single strand of suture in the muscle layer of back region in mice with intact skin, respectively, immediately and 5 d after operation in **b** (excitation wavelength: 808 nm; emission wavelength: 1000 nm; exposure time: 3000 ms). The dashed line in **a** and **b** correspond to the location of cross-sectional fluorescence intensity profiles for NIR-II. **c**, The decreased fluorescence intensity due to the dynamic process of suture degradation. Scale bar: 10 mm.

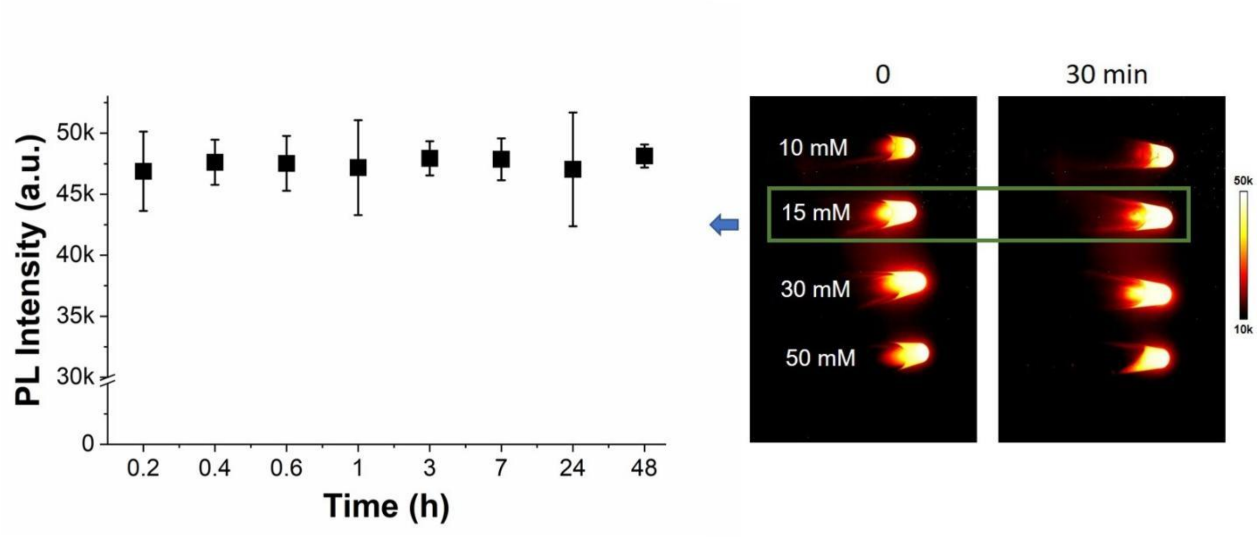

Supplementary Fig. 21 | The NIR-II fluorescence stability of BV-4B in n-BCA with different concentrations. The concentrations respectively 10, 15, 30 and 50 mM. All results presented as the mean ± s.d from n = 5 independent experiments.

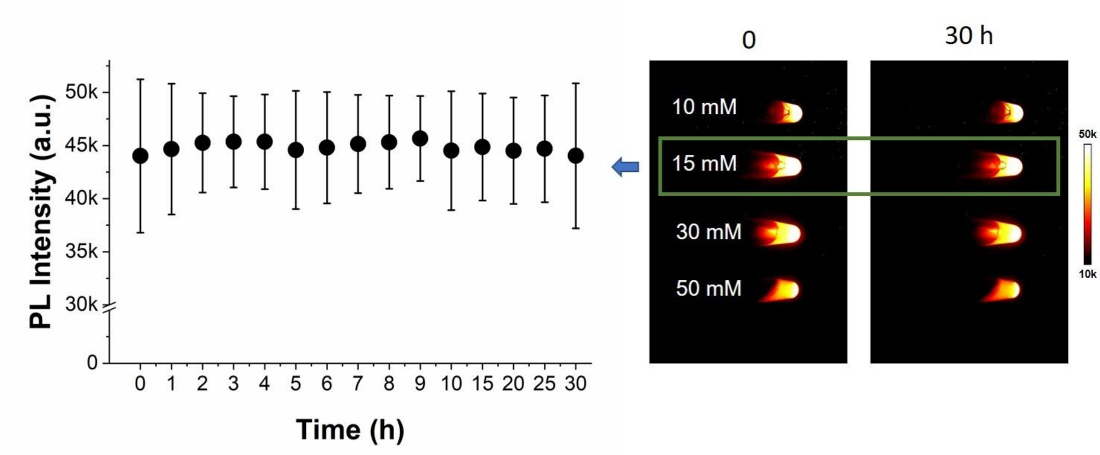

Supplementary Fig. 22 | The NIR-II fluorescence photostability of the complexes including BV-4B and n-BCA exposed to continuous 808 nm laser irradiation for 30 h. The concentrations respectively 10, 15, 30 and 50 mM in 100 µL of n-BCA. Excitation wavelength: 808 nm; emission wavelength: 1000 nm; exposure time: 1500 ms. All results presented as the mean ± s.d from n = 5 independent experiments.

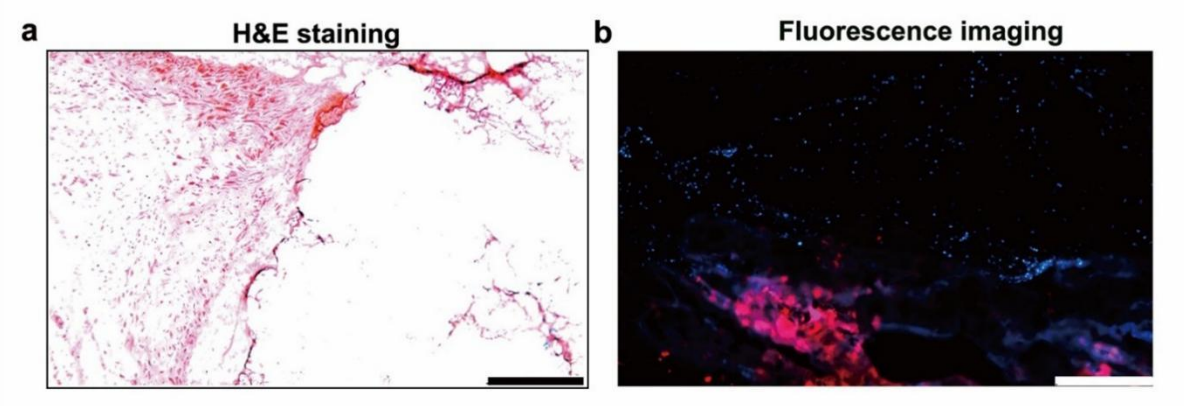

Supplementary Fig. 23 | Microscopic examination of the resected tissue from the arteriovenous malformation (AVM) nidus of the rat. a, The abnormal vessel wall which is in accordance with the AVM diagnosis by the hematoxylin and eosin (H&E) staining of the resected tissue. **b,** Fluorescence imaging of the embolization site. DAPI (blue), BV-4B (red, excitation wavelength: 542-582 nm; emission wavelength: 604-644 nm). Scale bar: 200 µm.

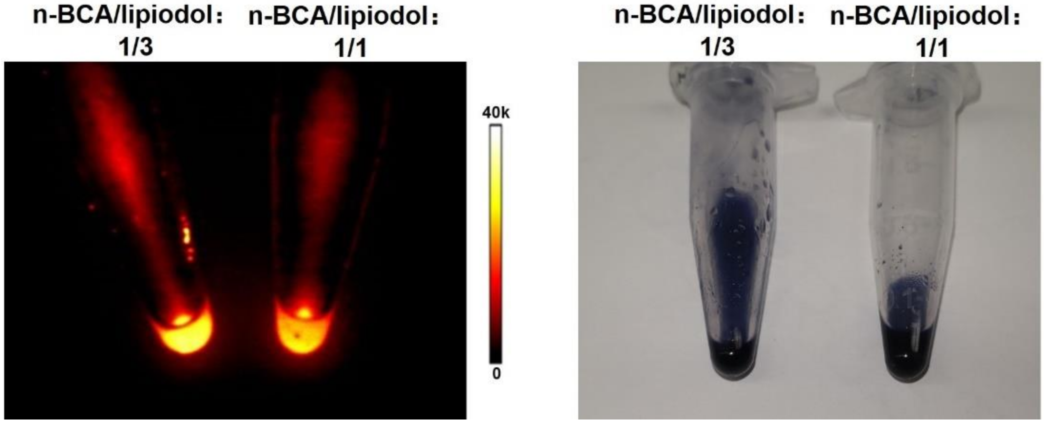

Supplementary Fig. 24 | No change of the NIR-II fluorescence from the suspension with n-BCA and BV-4B (30 mM) after adding lipiodol (ethiodized oil), as a radio-opaque contrast agent.

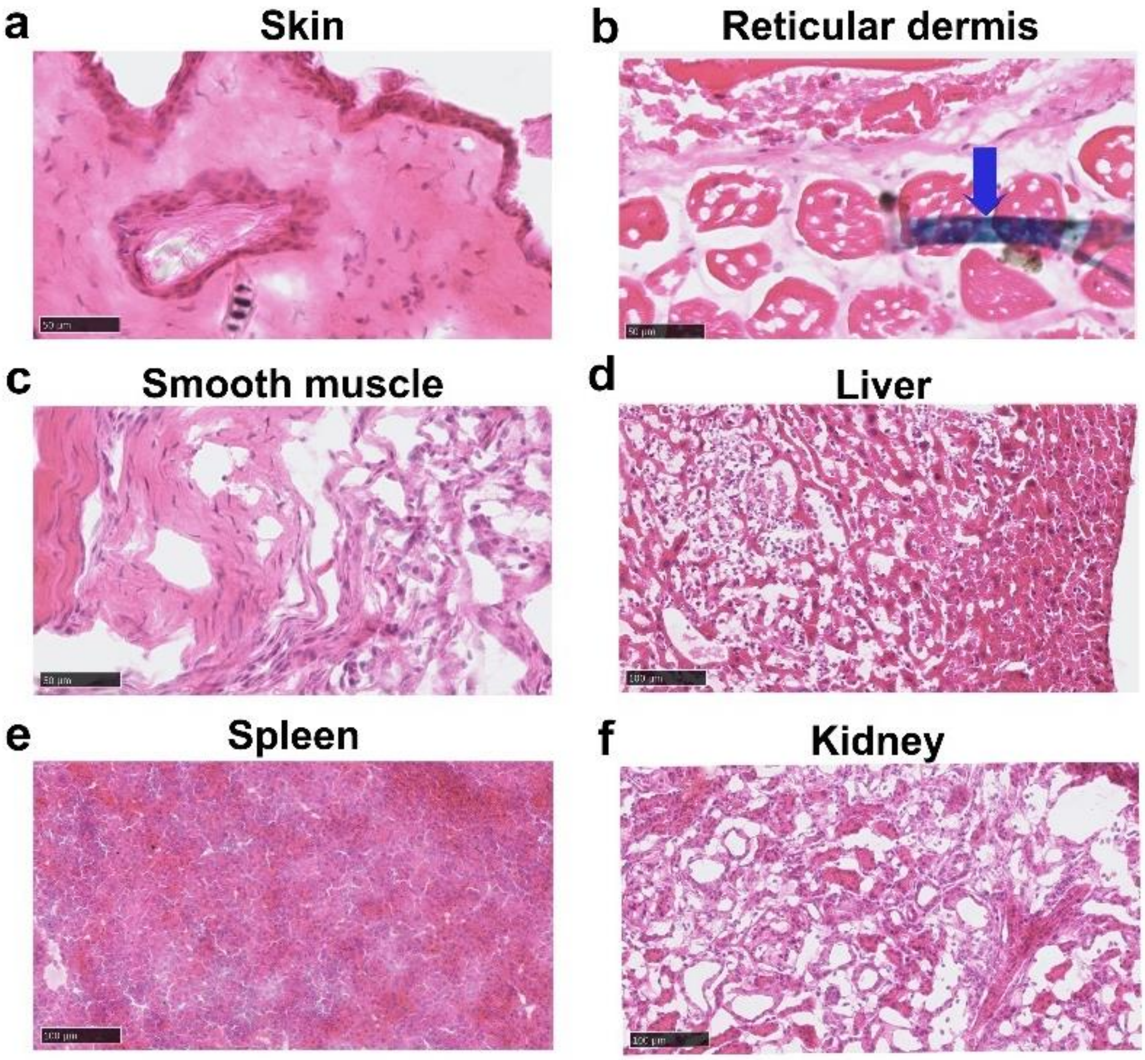

Supplementary Fig. 25 | Tissue toxicity analysis based on histologic staining on soft tissue around catheters and main organs. **a,** The normal layers of epidermis in skin by H&E staining. **b,** The normal reticular dermis with vessels and some collagen bundles. The longitudinal section of the BV-4B-coated silicone catheter (arrow). **c,** The skeletal and smooth muscle underneath the catheter. **d,** The liver, **e,** the spleen and **f,** the kidney with normal histologic appearance. Scale bar: (**a**-**c**) 50 µm; (**d**-**f**) 100 µm.

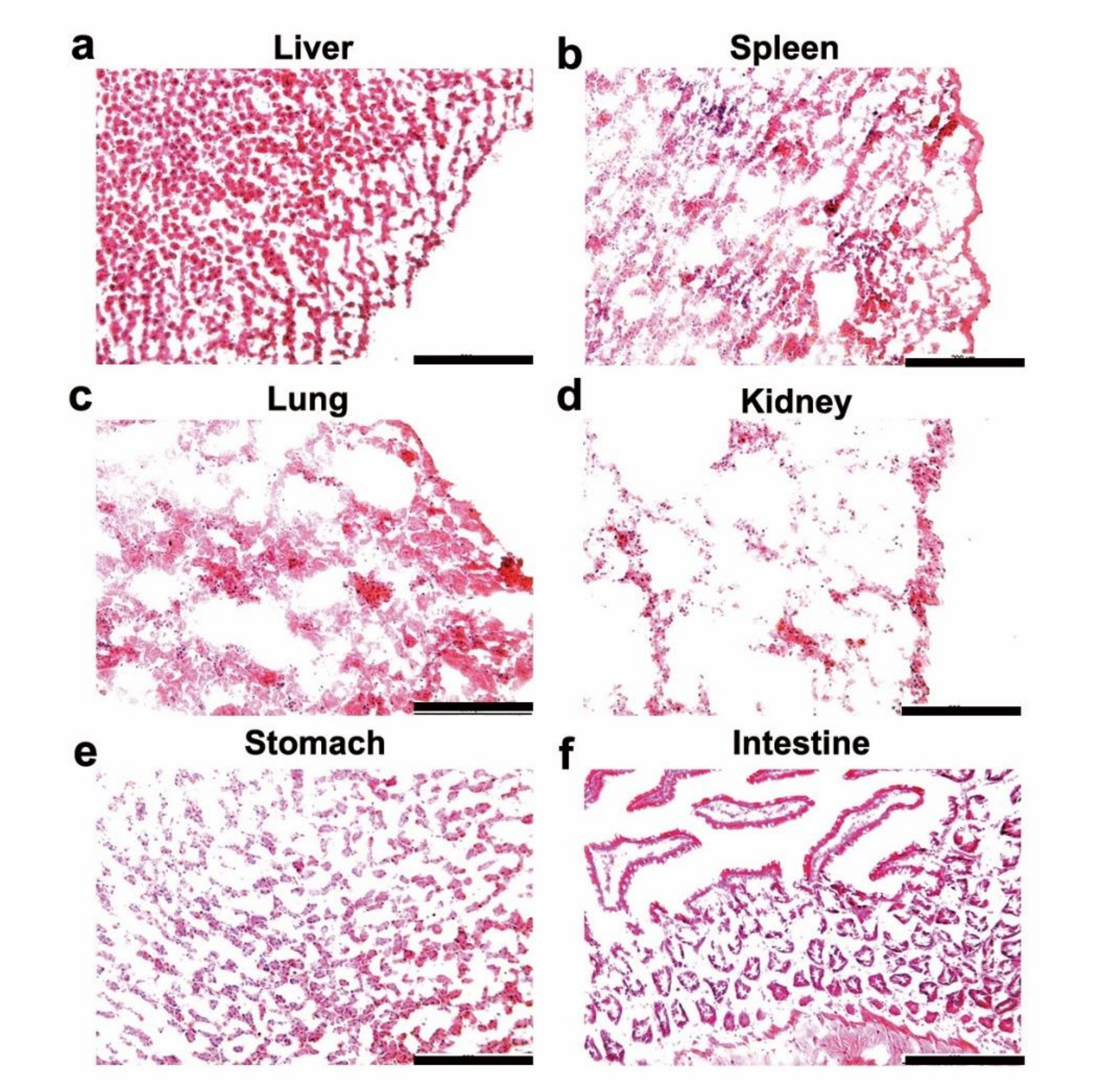

Supplementary Fig. 26 | Tissue toxicity analysis based on histologic staining of the main organs from the rat with arteriovenous malformation (AVM) which receive the hybrid fluorescence-guided surgery. **a,** Liver; **b,** Spleen; **c,** Lung; **d,** Kidney; **e,** Stomach; **f,** Intestine. Scale bar: 200 μm.

Supplementary Table 1 | The solubility of BV-4B.

T = 19 ^{o}C; Humidity = 55%.

Supplementary Table 2 | The dihedral angles between a-b, a-c and b-c of BV-4B in different solvents.

**Solvent**	**∠a-b (°)**	**∠a-c (°)**	**∠b-c (°)**
**Methanol**	56.26267	50.86826	57.09774
**THF**	52.28180	55.14638	54.67344
**Water**	53.05511	54.07216	54.10610
**Acetonitrile**	55.76933	49.96502	58.71218

**Sequence number**	**1**	**2**	**3**	**4**	**5**	**6**	**7**
**The mass of BV-4B /mg**	1.0	1.0	1.0	1.0	1.0	1.0	1.0
**Solvent**	DMSO	DMF	H_2O	Methanol	Ethanol	Isopropanol	n-Butanol
**Volume/ul**	7	4	17	5	43	315	233
